# Resolving Difficult Phylogenetic Questions: Why More Sequences Are Not Enough

**DOI:** 10.1371/journal.pbio.1000602

**Published:** 2011-03-15

**Authors:** Hervé Philippe, Henner Brinkmann, Dennis V. Lavrov, D. Timothy J. Littlewood, Michael Manuel, Gert Wörheide, Denis Baurain

**Affiliations:** 1 Département de Biochimie, Centre Robert-Cedergren, Université de Montréal, Montréal, Québec, Canada; 2 Department of Ecology, Evolution, and Organismal Biology, Iowa State University, Ames, Iowa, United States of America; 3 Department of Zoology, The Natural History Museum, London, United Kingdom; 4 Université Paris 6, UMR 7138 "Systématique, Adaptation, Evolution" UPMC CNRS IRD MHNH, Paris, France; 5 Department of Earth and Environmental Sciences, Ludwig-Maximilians-Universität München, München, Germany; 6 GeoBio-Center, Ludwig-Maximilians-Universität München, München, Germany; 7 Unit of Animal Genomics, GIGA-R and Faculty of Veterinary Medicine, University of Liège, Liège, Belgium; Massey University, New Zealand

In the quest to reconstruct the Tree of Life, researchers have increasingly turned to phylogenomics, the inference of phylogenetic relationships using genome-scale data (Box 1). Mesmerized by the sustained increase in sequencing throughput, many phylogeneticists entertained the hope that the incongruence frequently observed in studies using single or a few genes [1] would come to an end with the generation of large multigene datasets. Yet, as so often happens, reality has turned out to be far more complex, as three recent large-scale analyses, one published in *PLoS Biology* [2]–[4], make clear. The studies, which deal with the early diversification of animals, produced highly incongruent (Box 2) findings despite the use of considerable sequence data (see Figure 1). Clearly, merely adding more sequences is not enough to resolve the inconsistencies.

**Box 1. From Phylogenetics to Phylogenomics**

Phylogenetics, the determination of evolutionary relationships among organisms, is central to our understanding of the evolution of life. For instance, the three phylogenies of Figure 1 entail profoundly different interpretations about the complexity of the common ancestor of all animals. Important body plan characters (e.g., neurosensory and digestive systems and muscle cells) are found in cnidarians, ctenophores, and bilaterians but not in sponges and placozoans. According to the phylogenies of Schierwater et al. [4] and Dunn et al. [2], the taxonomic distribution of these characters implies either (i) that the ancestral metazoan already featured these traits and that sponges (and placozoans) have secondarily lost them or (ii) that these characters were acquired several times independently by convergence (e.g., in the cnidarian + ctenophore and in the bilaterian lineages, according to the tree of Figure 1A). In contrast, the phylogeny of Philippe et al. [3] is more congruent with morphological characters and compatible with a simple metazoan ancestor and a later emergence of these characters only once, in the lineage leading to the common ancestor of coelenterates (cnidarians+ctenophores) and bilaterians.

Phylogenies are generally depicted as trees (which are non-reticulated graphs, as in Figure 1) because vertical evolution is undisputedly the primary mechanism of inheritance for genetic material. However, the existence of horizontal transmission (e.g., hybridization of closely related taxa, organelle acquisition through endosymbiosis and horizontal gene transfer) makes phylogenetic trees only pragmatic approximations, which will probably be replaced by phylogenetic networks in the long term (particularly for unicellular organisms).

Recently, phylogenomics, the use of genomic data to infer evolutionary relationships, has emerged as a new domain of phylogenetics. The main strength of phylogenomics is the drastic reduction in random (or sampling) error brought by the use of large (multigene) datasets. Numerous approaches can be used to take advantage of genomic data (for review see [49]). Briefly, new methods based on oligonucleotide content, gene content, or intron positions look promising (as shown by their ability to yield reasonable trees) but require additional theoretical developments to achieve their full potential. That is why the two most popular phylogenomic approaches are simple extensions of the standard phylogenetics methods applied to single-gene datasets. The first, known as the “supermatrix” (or superalignment), consists in concatenating numerous orthologous genes into a single supergene, which is analyzed using standard methods (or slightly modified methods such as separate models allowing for multiple sets of branch lengths [50]). The second, “supertree,” approach takes the opposite path by first inferring a tree for each gene in the dataset and then combining these individual trees into a single supertree. The supermatrix approach is the most commonly used, in agreement with the handful of studies suggesting that it offers greater accuracy than the supertree [13],[51], though this remains to be formally demonstrated.

**Box 2. Glossary**

**Homology/orthology/paralogy/xenology:** Genes that derive from a common ancestor are termed homologs. Two homologous genes are orthologous if they diverged through a speciation event. In contrast, paralogs originate by duplication of a single gene within a given lineage, whereas xenologs result from the horizontal transfer of a gene from a donor species to a receiver species (which might eventually get its original copy replaced by the xenolog).

**Homoplasy/convergence:** Spurious similarity due to convergence or reversion and not to common ancestry is termed homoplasy. Convergence describes the independent acquisition by separate evolutionary lineages of the same nucleotide (or amino acid) at a given position. This is a direct consequence of multiple substitutions.

**Incomplete lineage sorting:** The transient retention of ancestral polymorphisms across speciation events. Speciations compressed in time and large reproductive populations both increase the likelihood of this phenomenon. Considering three lineages having rapidly diverged, by chance some sequence positions will be shared between one pair, while others will be shared between another pair, and yet others between the third possible pair, hence blurring the phylogenetic signal on the corresponding branches.

**Incongruence:** Two (or more) phylogenetic trees are said to be incongruent when they exhibit conflicting branching orders (i.e., topologies) and cannot be superimposed. This implies that at least one node (also known as a bipartition) present in one tree is not found in the other(s), where it is replaced by alternative groupings of taxa.

**Model of sequence evolution:** A statistical description of the process of substitution in nucleotide or amino acid sequences. Complex models better approximate the evolutionary process but at the expense of more parameters and computational time. As parameter-rich models require more data to behave properly, they have become really useful with the advent of phylogenomic datasets.

**Monophyly:** To be considered monophyletic, a taxonomic group must satisfy two conditions: (i) all its taxa must derive from a single ancestor and, reciprocally, (ii) all taxa deriving from this common ancestor must belong to the group.

**Non-phylogenetic signal:** The combination of different kinds of structured noise (e.g., undetected homoplasies) that compete with the genuine phylogenetic signal during tree reconstruction. Even if the non-phylogenetic content is partly a property of a multiple sequence alignment (notably related to its saturation level), the non-phylogenetic signal actually inferred heavily depends on the method and the model of evolution selected. In probabilistic methods, the non-phylogenetic signal mainly results from the data violating the model of sequence evolution. These violations arise because our models are inevitably oversimplified in comparison to the complexity of the natural evolutionary process. Eventually, the apparent signal analyzed will be a blend of phylogenetic and non-phylogenetic signal.

**Outgroup/ingroup:** Nearly all tree reconstruction methods produce unrooted trees, in which inferred relationships do not convey any information about the direction of time. To root a tree and turn it into a phylogeny, one has to include in the analysis a group of taxa that are known to be outside the group under study. This reference group is termed the outgroup, while the taxa of interest make the ingroup.

**Patristic distance:** The sum of the lengths of the branches that connect two nodes in a phylogenetic tree, where those nodes are typically terminal nodes representing extant taxa. It is thus an inferred distance (taking into account multiple substitutions) greater than the uncorrected distance directly computed from the number of differences observed between the two corresponding sequences in the alignment.

**Phylogenetic signal/synapomorphy:** The substitutions occurring along a given branch of the evolutionary tree. The strength of the phylogenetic signal is proportional to the number of substitutions occurring along the branch. In non-probabilistic methods, the signal is encoded in synapomorphies, i.e., shared residues (nucleotides or amino acids) at aligned positions that are specific to a set of sequences derived from a common ancestor. In probabilistic methods, the amount of phylogenetic signal actually extracted from a given dataset depends on the model and is expected to increase with the fit of the model to the data (i.e., the ability of the model to explain the data).

**Phylogenetic tree:** A (connected acyclic) graph describing the estimated evolutionary relationships among a group of species. In molecular trees, branch lengths are proportional to the genetic distances (and hence to some extent to time) inferred from the analysis of a multiple alignment of homologous sequences (nucleotide or amino acid sequences).

**Probabilistic methods:** A family of tree reconstruction methods from multiple sequence alignments that are grounded in statistical theory and make use of explicit models of sequence evolution. These include maximum likelihood and Bayesian inference approaches and are known to be the most accurate but also the most computationally demanding.

**Saturation:** When sequences in a multiple alignment have undergone so many multiple substitutions that apparent distances largely underestimate the real genetic distances, the alignment is said to be saturated. Phylogenetic inference works best with datasets that are only slightly saturated. Owing to their reduced state space (four possible bases), nucleotide sequences saturate more rapidly than protein sequences (20 possible amino acids).

**Site-homogeneous/site-heterogeneous models:** Most models of sequence evolution assume that the same evolutionary process takes place at every position (or site) of an alignment. With such models, only the evolutionary rate can be modeled as heterogeneous across sites, usually through a gamma distribution of rates. However, selective constraints are known to be quite heterogeneous across positions, hence seriously violating the hypotheses of site-homogeneous models. On the other hand, site-heterogeneous models assume that the evolutionary process varies widely across sites, in particular the set of acceptable amino acids (e.g., in the CAT model). A number of studies have demonstrated that site-heterogeneous models provide a better fit to phylogenomic datasets and tend to reduce the sensitivity to tree reconstruction artifacts (e.g., LBA).

**Figure 1:**
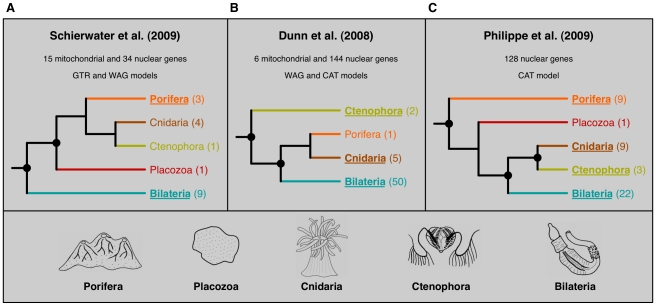
Simplified representation of the trees obtained in three recent phylogenomic analyses of early animal diversification. (A) Schierwater et al. [4] tree. (B) Dunn et al. [2] tree. (C) Philippe et al. [3] tree. Numbers in parentheses after taxon names indicate the number of species included in the dataset for the corresponding taxon. Bootstrap support values above 90% are indicated by a bullet (for nodes) or by underlining (for terminal taxa). It is worth mentioning that the monophyly of Porifera is not unequivocally accepted [28],[46]; only the analysis of 30,000 positions with a rich taxon sampling and a complex model of evolution recovers it with significant statistical support [3]. Although such a sparse phylogenetic signal will require harnessing the full potential of phylogenomics to be confidently solved, this question is outside the scope of this study. Simplified drawings (redrawn from [74]) on the bottom illustrate the huge morphological disparity existing between the five terminal taxa. Porifera correspond to sponges; Cnidaria to sea anemones, jellyfishes, and allies; Ctenophora to comb jellies; and Bilateria to all other animals (characterized by their bilateral symmetry) except *Trichoplax* (Placozoa), which appears to be morphologically the most simply organized animal phylum.

Here, taking these three studies as a case in point, we discuss pitfalls that the simple addition of sequences cannot avoid, and show how the observed incongruence can be largely overcome and how improved bioinformatics methods can help reveal the full potential of phylogenomics.

## Hurdles to Phylogenomics

Two factors contribute significantly to the difficulty of reconstructing the correct phylogenetic tree for a set of sequences. First, if speciation events are closely spaced in time, the amount of phylogenetic signal is often small, leading to short internal tree branches that are difficult to resolve [5],[6]. Second, if the events of interest are ancient, terminal branches tend to be long and replete with multiple substitutions occurring at the same position (i.e., homoplasy). In the extreme case, insufficient signal may remain for very deep divergences to be resolved even when using very long gene sequences [7]—but this issue is outside the scope of the present contribution. Depending on the accuracy of the model of sequence evolution, multiple substitutions can go undetected or be wrongly inferred. In both situations spurious phylogenetic signals are generated; these constitute the major part of what we collectively term non-phylogenetic signal. The best known example of the misleading effect of non-phylogenetic signal is the long branch attraction (LBA) artifact [8]: when two (or more) lineages have much longer branches than the others, they tend to group together irrespective of their true relationships. Notably, the outgroup is a natural source of long branches that may attract fast-evolving (hence long branched) species of the ingroup. When this happens, attracted branches artifactually emerge too deeply in the tree [9].

Inferring phylogenies in difficult cases is akin to finding a needle (phylogenetic signal) in a haystack. Under the oversimplified assumption of an absence of non-phylogenetic signal, one can compute that the resolving power would increase from approximately 15 million years when using small subunit ribosomal RNA alone to less than 1 million years when using more than 50 genes [10]. At such levels of resolution, incomplete lineage sorting (i.e., the retention of ancestral polymorphisms over successive speciation events) should be taken into account as a potential source of phylogenetic error [11]. Nonetheless, even if conflicting gene genealogies were not an issue, throwing additional gene sequences at a difficult phylogenetic question does not necessarily solve the problem—the size of the needle is indeed increased, but so too is the size of the haystack. It follows that non-phylogenetic signal may become dominant and yield incongruent, yet statistically highly supported, phylogenomic trees [12].

## How to Prevent Deleterious Effects of Non-Phylogenetic Signal

Non-phylogenetic signal has multiple and disparate sources [13]. When multiple genes are concatenated and analyzed with standard methods (but see [14]), non-phylogenetic signal is caused by the inclusion of sequences that deviate from the true species phylogeny or by the inability of our methods to correctly handle multiple substitutions. In practice, it mainly stems from (i) the incorrect identification of orthologs, (ii) erroneous alignments, or (iii) the incorrect reconstruction of multiple substitutions occurring at a given position, the last owing to model violations in probabilistic methods (i.e., Bayesian inference and maximum likelihood). Although all three aspects have received considerable attention from theoreticians, and despite the availability of numerous bioinformatics tools [15]–[17], there is still no magic bullet. That is why classic phylogenetics involves numerous refinements and controls, which are difficult, but not impossible, to apply at a phylogenomic scale.

Non-phylogenetic signal can be reduced by improving (i) the quality of primary alignments through selection of the orthologous genes that are least subject to saturation and (ii) the detection of multiple substitutions, which is best achieved by using both a large number of species and the most realistic model of sequence evolution. In the following, we show that both improvements are required at the same time to address the difficult question of the relationships among major animal groups, i.e., sponges, placozoans, ctenophores, cnidarians, and bilaterians. Reanalysis of the underlying data indicates that failure to apply one or more of the strategies intended to decrease non-phylogenetic signal is what caused the incongruent, though strongly supported, results that were recently observed [2]–[4].

## Issues at the Level of Sequence Alignments

Selection of unambiguously orthologous genes [18] is usually achieved by targeting single-copy genes (e.g., mitochondrial genes) or pre-selected genes (e.g., ribosomal RNAs and proteins), or through automatic clustering methods. None of these options are without problems. Both manual and automatic methods [19]–[22] heavily rely on BLAST similarity scores, which are known to be a poor estimator of the true evolutionary distance [23]. Given the limitations of existing methods of orthology detection (Box 3), careful phylogenetic analysis of each alignment is important to achieve maximal accuracy. However, this manual step is difficult and subjective. That is why it is preferable to also verify orthology a posteriori. One possibility is to assess whether branches receiving high statistical support from every single gene tree are congruent with the species tree [18]. Though the latter is unknown, the phylogeny obtained by the concatenation of numerous genes constitutes a reasonable approximation. Hence, Philippe et al. [3] looked at every supported branch (bootstrap support [BS]≥70%) from single-gene trees that were incongruent with the concatenated tree to assess the orthology of their pre-selected genes. Only 6.5% of the branches were incongruent, and almost all conflicts were best explained by reconstruction errors affecting single-gene trees [3]. According to this semi-automated approach, the 128 genes used in [3] can be provisionally considered as orthologous and suitable for phylogenetic analysis. In contrast, when applied to the datasets of Schierwater et al. [4] and Dunn et al. [2], the very same approach identifies several instances of incongruence between single-gene and concatenated trees (mainly apparent horizontal gene transfers that are in fact more likely due to contaminations, or deep unrecognized paralogy; see Text S1 and Figures S1, S2, S3, S4, S5, S6, S7, S8, S9).

**Box 3. Quality Control of Phylogenomic Datasets**

Despite the great progress in software development [19]–[22],[52]–[54], our nine years of experience with large-scale multigene analyses [55] leads us to conclude that computer-assisted manual expertise is not yet dispensable. In particular when processing EST data, two issues are still challenging to handle by automation: (i) the non-homology of short sequence stretches due to frameshifts and point mutations and (ii) the non-orthology of one or more genes with similar sequence for some species, because of paralogy or xenology, along with taxonomic misidentifications and library contaminations (e.g., by parasites such as platyhelminthes). An important limitation of automated methods for checking single-gene alignments for orthology prior to concatenation is the limited amount of sequence information available in a single gene, which often makes current statistical analyses impractical. If the threshold used is stringent, almost every sequence will fail the test, whereas a loose threshold will lead to numerous false positives. Manual verification, through visual inspection of alignments and phylogenies, can to a large extent compensate for this lack of statistical power if a large number of species (much more than those eventually included in the final analysis) is taken into account. First, as conserved positions are clearly identified, both translational frameshifts (leading to stretches of amino acids highly different from the consensus, which are mostly found at EST extremities) and local sequencing errors (visible as unmatched amino acids at highly conserved positions) stand out. Based on manual analysis, we estimate that approximately 4,800 amino acids (0.66% of the complete alignment) were erroneous in the Dunn et al. dataset [2] because of frameshifts and local sequencing errors (including incorrect translation owing to a mistake in the specification of the genetic code for ambulacrarian mitochondria; see Table S2). Second, xenology, contaminations, and misidentification can be efficiently detected when individual alignments encompass a broad taxonomic diversity, as such diversity is much more likely to find a close relative of the donor species. For instance, in the Dunn et al. dataset [2], one acoel species, the marine flatworm *Neochildia fusca*, was contaminated by microsporidia (see Table S2). Since original alignments lacked microsporidial sequences, the contamination was overlooked and acoel sequences were simply considered as extremely divergent. Similarly, hidden paralogy is easier to detect with numerous species on hand (and with deeper sequencing of each of them), because they increase the chance of finding a species that has kept both copies. Interestingly, much more serious errors (including the use of paralogous, rather than orthologous, copies, and taxonomic misidentification; see Figures S1, S2, S3, S4, S5, S6, S7, S8, S9) were identified in the manually assembled Schierwater et al. dataset [4] than in the automatically assembled Dunn et al. dataset [2] (compare Tables S1 and S2). Manual assessment of the quality of primary data is particularly tedious and time-consuming, as well as error-prone. That is why automated approaches featuring refined statistics (e.g., hidden Markov models detecting frameshifts) are strongly needed to both speed up and improve the construction of phylogenomic datasets. Finally, it should be noted that missing data (i.e., incomplete sequences), which are on the rise in recent large-scale analyses (e.g., 55.5% of the characters in [2] and 81% in [46]), constitute an additional unpredictable issue, as they might further erode statistical power and sometimes enhance tree reconstruction artifacts [38],[42] (see Text S1 and Figure S11).

**Figure 2:**
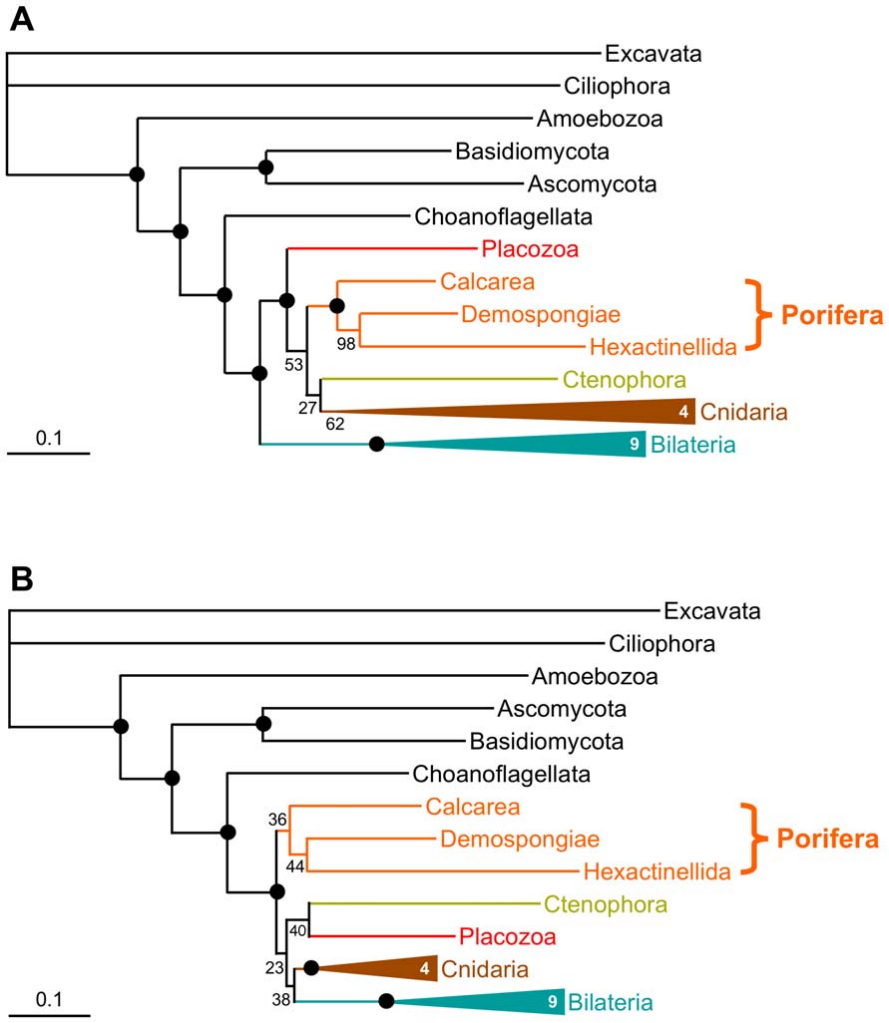
Analysis of the revised Schierwater et al. dataset. (A) Scheme of the original tree [4]. (B) Scheme of the tree obtained with the revised dataset. Both trees were inferred using exactly the same probabilistic method and model (i.e., using RAxML [75] with a GTR+Γ model for nucleotide sequences and a LG+F+Γ model for protein sequences). Numbers in the triangles indicate the number of species used for the corresponding clade. Bullets denote maximum bootstrap support values (BS = 100%); lower values are given. In the revised dataset, numerous discrepancies were corrected (Table S1), and a few genes were discarded because of dubious orthology; 14,112 unambiguously aligned positions were retained. Furthermore, the erroneous use of mitochondrial sequences of demosponge origin to represent both hexactinellids and calcareans (Figure S9) in the original study [4] drastically—yet probably artifactually—strengthened the support for the monophyly of sponges (BS = 100%; [A]), whereas it appeared much weaker in our reanalysis (BS = 36%; [B]), in line with previous studies [24],[26]–[28] that failed to find significant support for or against sponge monophyly (but see [3]). See Figure S10 for the complete tree obtained with the revised dataset.

This as well as the discovery of other important issues (see Table S1) prompted us to reassess and reanalyze the dataset of Schierwater et al. [4]. The revised phylogeny we generate (Figure 2B) differs from the original one (Figure 2A) in the deep animal relationships: the strong support for a sister-group relationship between Bilateria and a group composed of placozoans, sponges (Porifera), ctenophores, and cnidarians [4] has vanished, and sponges are now recovered as the sister group of all other Metazoa. Strikingly, this part of the revised tree (Figure 2B) suffers from a lack of statistical support (all BS<50% except for the monophyly of cnidarians). The simplest explanation for these results (Figure 2B) is that the genuine phylogenetic signal for non-bilaterian animal relationships is scarce, as reported in all previous studies (e.g., [24]–[28]). The possible inclusion of non-orthologous sequences (see Figures S1, S2, S3, S4, S5, S6, S7, S8, S9) might create a strong signal that could overcome the genuine but faint phylogenetic signal, and lead to the incorrect—but strongly supported—monophyly of “diploblasts” (sponges+placozoans+ctenophores+cnidarians) that was observed in the original study (Figure 2A). Otherwise, the topology we infer from the revised alignments is similar to the published tree [4], with only three nodes differing out of 21. This demonstrates that phylogenomics is relatively robust to the possible inclusion of non-orthologous sequences when the genuine phylogenetic signal is abundant (see also [29],[30]), which can be explained by the randomness of most of the introduced errors preventing the appearance of a structured misleading signal.

On the other hand, phylogenomics is sensitive to the non-phylogenetic signal that stems from the incorrect inference of multiple substitutions. By devoting a large part of their dataset to mitochondrial genomes, which are fast-evolving in Bilateria (e.g., [24],[31]), Schierwater et al.'s solution unwittingly favored the emergence of Bilateria between the outgroup and a group composed of all the non-bilaterian Metazoa, because of the LBA artifact. This artifact probably also affects the phylogeny of Dunn et al. [2]; in that case, the fast-evolving ctenophores are likely attracted by the distant outgroup (see Text S1). In the phylogeny inferred from an updated version of the alignments of Dunn et al. (purged of several sequencing errors and species misidentifications—see Table S2—and completed with new sequences, thereby reducing the amount of missing data from 55% to 35%), sponges are the sister group of all other Metazoa, with the fast-evolving Ctenophora representing the sister group of Cnidaria plus Bilateria (Figure S11; see also [32]).

In summary, analyzing the revised alignments from Schierwater et al. [4] and Dunn et al. [2] with their original taxon sampling and inference methods is sufficient to eliminate all significant incongruences among the three recent phylogenomic studies (Figure 1). The variability in robustness across the tree (e.g., Figure 2) underscores the importance of clean phylogenomic datasets: whereas large amounts of phylogenetic signal usually drown out any non-phylogenetic signal, for nodes characterized by a scarce phylogenetic signal, even small amounts of non-phylogenetic signal may dominate and eventually yield incorrect results [10].

## Issues at the Level of Taxon Sampling

**Figure 3:**
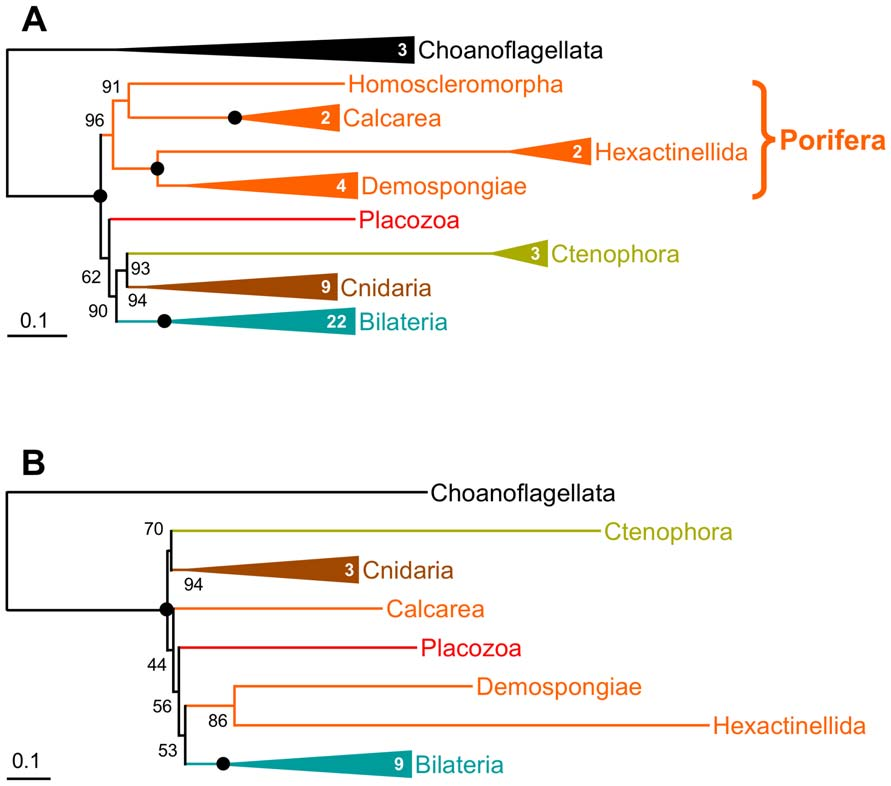
Reanalysis of the Philippe et al. dataset with a reduced taxon sampling. (A) Scheme of the original tree [3]. (B) Scheme of the tree obtained after reduction of the taxon sampling. Both trees were inferred using exactly the same probabilistic method and model (i.e., PhyloBayes using the CAT+Γ model [76]). Numbers in the triangles indicate the number of species used for the corresponding clade. Bullets denote maximum bootstrap support values (BS = 100%); lower values are given. See Figure S12 for the complete tree obtained after reduction of the taxon sampling.

The lack of support observed in Figures 2 and S11 contrasts with the high bootstrap values obtained by Philippe et al. [3] for the monophyly of each of the Porifera (96%), Coelenterata (Cnidaria+Ctenophora, 93%), and Eumetazoa (all animals except Porifera and Placozoa, 90%) (Figure 3A). However, the number of non-bilaterian metazoan species used in [3] is larger, 22 versus 9 [2],[4], which could account for the difference. Indeed, it is well known that including more species allows for a better detection of multiple substitutions [33], as it decreases the amount of non-phylogenetic signal while preserving phylogenetic signal [34]; this is why authors often mention that their results should be viewed as provisional until more taxa are considered (e.g., the position of Ctenophora in [2]). To test this hypothesis, we reduced the taxon sampling of [3] to match as closely as possible the sampling of Figure 2. Even though sequences and inference methods are exactly as in [3], the support for deep animal relationships decreases drastically (Figure 3B). While the monophyly of each of the Cnidaria (94%), Coelenterata (70%), and Demospongiae + Hexactinellida (86%) still receive some support, remaining relationships are unresolved (BS<60%); in particular, Porifera and Eumetazoa are not recovered. These results corroborate the hypothesis that the use of a limited number of species generates enough non-phylogenetic signal to swamp most of the faint genuine phylogenetic signal present in this part of the animal phylogeny (owing to short internal branches and heterogeneous rates among species).

However, taxon sampling is not simply a matter of number of species [35]–[37]. In particular, the inclusion of both slowly evolving species and closely related outgroups (e.g., choanoflagellates for animals; see [3] and Text S1) is often of prime importance. This point is well illustrated by a reanalysis of the original alignments of Schierwater et al. in which we eliminated the most distant outgroups. When rooting exclusively with choanoflagellates, the bootstrap support for a position of Porifera as the sister group to remaining animals rises to 80% (Figure S13). Although discarding very distant outgroups (e.g., Bacteria) undoubtedly improves accuracy, the effect of including moderately distant outgroups (e.g., Fungi) in addition to close outgroups (e.g., choanoflagellates) is more difficult to assess. Eventually, it will depend on the relative influence of introducing a very long branch (the distant outgroup) and breaking up an already existing long branch (the close outgroup). Even if further studies are needed to clarify this point, an effort to increase the taxon sampling of the close outgroup should help to resolve deep animal relationships.

Finally, phylogenomic datasets, especially when based on expressed sequence tag (EST) data, are frequently characterized by incomplete gene coverage for some taxa. Yet, there have been few attempts to determine whether missing data per se can cause errors in tree reconstruction [36],[38]–[42] and how they may interfere with other aspects of phylogenetic inference. In particular, it is not known whether a smaller, but complete, alignment of targeted genes (e.g., selectively amplified by PCR) would yield a more accurate and robust tree than a large, but incomplete, alignment of highly expressed genes (obtained by EST sequencing). These questions can and should be better assessed in the near future.

## Issues at the Level of Tree Reconstruction Methods

**Figure 4:**
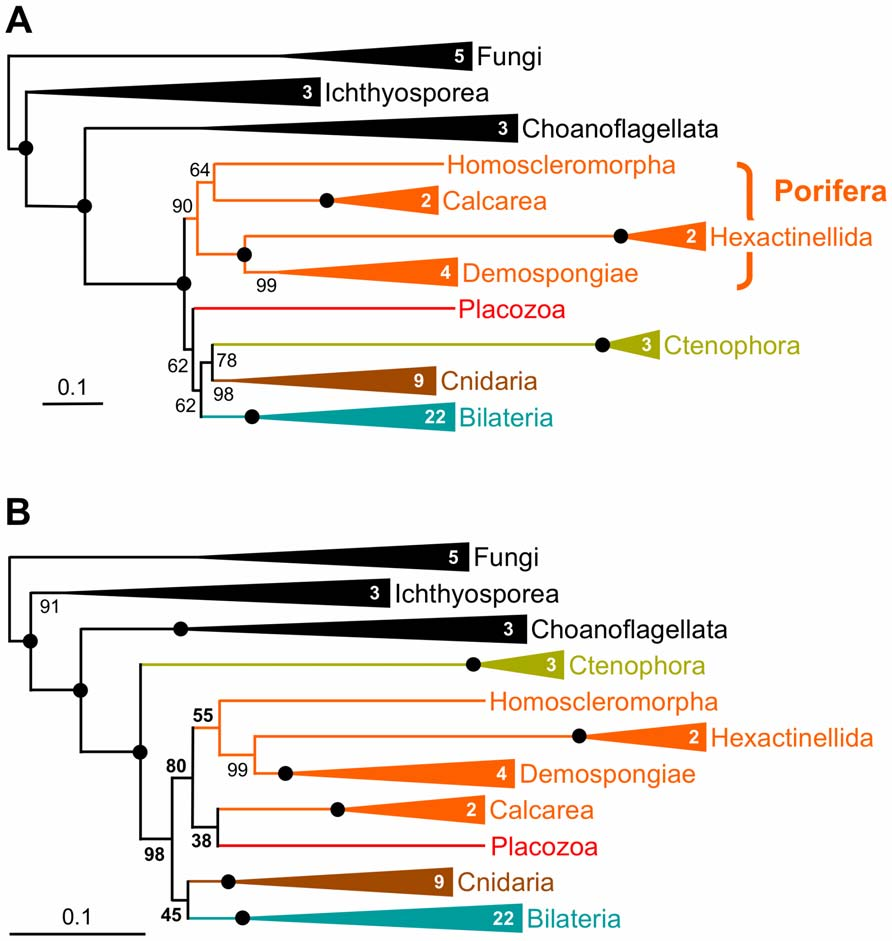
Reanalysis of the Philippe et al. dataset with a less complex model. (A) Scheme of the original tree [3] obtained with the CAT+Γ model. (B) Scheme of the tree obtained with the less complex WAG+F+Γ model. Both trees were inferred using exactly the same dataset. The WAG+F+Γ model has a less good fit to this alignment than the CAT+Γ model [3]. Numbers in the triangles indicate the number of species used for the corresponding clade. Bullets denote maximum bootstrap support values (BS = 100%); lower values are given. See Figure S14 for the complete tree obtained with the less complex WAG+F+Γ model.

To further explore the idea that the paramount issue in phylogenomics pertains to the reduction of non-phylogenetic signal (more than the increase of phylogenetic signal with datasets containing more and more genes, especially in the short run), we now turn to the selection of the model of sequence evolution. Since their origin [43], the main objective of these models has been to efficiently detect multiple substitutions (Box 4). We reanalyzed the dataset of [3] with a less accurate model, i.e., the site-homogeneous WAG+F+Γ model [44] used in [4] instead of the site-heterogeneous CAT+Γ model [45] used in the original study [3] (Figure 4A). In the WAG+F+Γ tree (Figure 4B), not only does resolution decrease (see BS of 43%, 45%, or 55%), but also the fast-evolving ctenophores now emerge at the base of all animals with strong support (BS = 98%), exactly as expected for a LBA artifact due to model mis-specifications. This indicates that when the less appropriate WAG+F+Γ model is used, multiple substitutions are so poorly inferred that branch lengths are miscalculated (i.e., non-phylogenetic signal has overwhelmed phylogenetic signal).

**Box 4. Improving Phylogenetic Inference Methods**

There is broad consensus on the necessity of using probabilistic methods in phylogenetic inference. Development of more accurate models of sequence evolution is central to the improvement of these methods. This generally implies more complex models, which are expected to come with increased computational load. Hence, in-depth analyses of datasets that are rich in both genes and species with such models can become prohibitive [46]. Consequently, some promising approaches, e.g., accounting for three-dimensional structure of proteins [56],[57] or performing joint alignment and phylogeny [58],[59], will probably stay out of reach for years. Fortunately, numerous recent algorithmic developments [60]–[62] significantly speed up phylogenetic computations, thus paving the way for model improvements. One generally considers that models should be biologically sound. Although biological realism is particularly important for understanding molecular evolution, it is less central for phylogenetic inference, where improving detection of multiple substitutions should be the top priority. As a result, models that more accurately distinguish a synapomorphy from a convergence greatly improve phylogenetic accuracy. Briefly, major steps forward were the modeling of heterogeneity of rate across species [63], heterogeneity of rate across substitutions [64],[65], heterogeneity of nucleotide/amino acid composition across species [66],[67], heterogeneity of rate across sites [68], and heterogeneity of the substitution process across sites [45]. In contrast, some other improvements, e.g., to handle heterotachy (i.e., heterogeneity of rate over time), had limited effects on phylogenetic reconstruction [69]; heterogeneity of rates across genes, handled by separate models [50], also has limited impact ([70], but see [71]). Future progress is expected (i) from the combination of various existing models [72], (ii) from the handling of other complexities, such as the heterogeneity of the substitution process over time, and (iii) from the handling of incomplete lineage sorting [11],[73].

In summary, the incongruence at the base of the animal tree observed in recent phylogenomic studies [2]–[4] can be explained by (i) a limited amount of phylogenetic signal, reflected in the short internal branches, and (ii) a profusion of confounding non-phylogenetic signal in certain cases. Since genuine phylogenetic signal is similar in all three analyses (i.e., internal branch lengths are identical and datasets are of similar size), conflicts are due to variations in the level of non-phylogenetic signal—depending on the quantity of non-orthologous sequences included, the number of species considered, and the model of sequence evolution selected. Ultimately, the ratio of phylogenetic to non-phylogenetic signal will determine the outcome: (i) when the phylogenetic signal is strong (sufficiently long internal branches), phylogenomics is always able to recover the correct topology, as found in the three studies [2]–[4] for outgroup and bilaterian phylogenies; (ii) when both signals are weak, results are statistically non-significant, as is often observed for deep animal relationships; and (iii) when the phylogenetic signal is weak (short internal branches) and the non-phylogenetic signal is strong (e.g., scarce taxon sampling), an artifactual topology is robustly inferred, such as the monophyly of “diploblasts” [4] or the basal emergence of ctenophores (Figure 4B) (see also [2],[32],[46]).

## Issues at the Level of Gene Sampling

**Figure 5:**
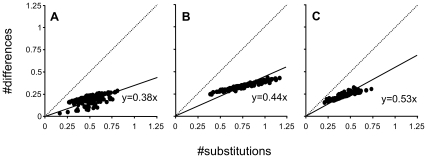
Saturation levels of datasets from Schierwater et al., Dunn et al., and Philippe et al. (A) Schierwater et al. [4] dataset. (B) Dunn et al. [2] dataset. (C) Philippe et al. [3] dataset. The revised alignments from Schierwater et al. and Dunn et al. were used (available as Datasets S1 and S2; see Text S1). The level of saturation was estimated for each dataset by computing the slope of the regression line of patristic distances (*y*-axis) versus uncorrected distances (*x*-axis), as previously described [12]. Patristic distances between two species were computed from branch lengths of the best maximum likelihood tree (using a GTR+Γ model for nucleotide sequences and a LG+F+Γ model for protein sequences).

Last but not least, it should be noted that not all genes contain the same potential amount of non-phylogenetic signal. Depending on both functional constraints and evolutionary trajectory, different genes can include positions subject to different ranges of multiple substitutions, i.e., they may display variable levels of saturation. To estimate the saturation in the three datasets [2]–[4], we used the comparison of patristic and uncorrected distances [47]. As shown by the slope of the regression line (data without any saturation have slope = 1; see [12]), the three datasets (Figure 5) are different, with that of Schierwater et al. being the most saturated (slope = 0.38) and that of Philippe et al. the least affected by multiple substitutions (slope = 0.53). This uneven amount of non-phylogenetic signal explains in part the differences observed in the three studies, but is difficult to separate from other factors. The phylogeny of Figure 1C, with the monophyly of each of Coelenterata (cnidarians+ctenophores) and Eumetazoa (all animals except sponges and placozoans), could be considered as the working hypothesis, because Philippe et al. [3] strived to minimize all three sources of non-phylogenetic signal (through the use of weakly saturated genes, a large number of species, and a complex model of sequence evolution). Nevertheless, the scarcity of phylogenetic signal shown here argues strongly for additional studies to confidently resolve the relationships among non-bilaterian animals.

## Conclusion

Contrary to common belief, some degree of conflict has to be expected when applying phylogenomics to difficult phylogenetic questions, because of the prevalence of non-phylogenetic signal. Consequently, we stress the necessity of reducing its impact. Since taxon and gene sampling is being rapidly improved by the relentless progress in sequencing technology (even if obtaining well preserved and correctly identified specimens remains the limiting factor for several key taxa), full achievement of the ultimate goal of phylogenomics—i.e., accurate resolution of the Tree of Life—will primarily hinge on better procedures for the selection of orthologous and least saturated genes as well as on improved models of sequence evolution. In summary, while we certainly encourage the inclusion of neglected groups of organisms in large-scale sequencing studies (e.g., [2],[3],[46],[48]), we consider at least as important that phylogeneticists engage in theoretical and bioinformatics developments that keep pace with sequencing technology to overcome these serious bottlenecks. This is essential to ensure that lessons learned from classical and molecular systematics are not forgotten in the phylogenomic era.

## Supporting Information

Dataset S1 Updated alignment of the Schierwater et al. dataset under the Nexus format.
(0.10 MB ZIP)

Dataset S2 Updated alignment of the Dunn et al. dataset under the Nexus format.
(0.46 MB ZIP)

Figure S1 Phylogeny of the AT6 gene.
(0.06 MB PDF)

Figure S2 Phylogeny of the CDC gene.
(0.06 MB PDF)

Figure S3 Phylogeny of the RP3 gene.
(0.06 MB PDF)

Figure S4 Phylogeny of the EF1 gene.
(0.04 MB PDF)

Figure S5 Phylogeny of the H70 gene.
(0.04 MB PDF)

Figure S6 Phylogeny of the PAX gene.
(0.04 MB PDF)

Figure S7 Phylogeny of the RAS gene.
(0.06 MB PDF)

Figure S8 Phylogeny of the CO2 gene.
(0.06 MB PDF)

Figure S9 Taxonomic misidentification for mitochondrial proteins of sponges.
(0.06 MB PDF)

Figure S10 Analysis of the revised Schierwater et al. dataset.
(0.04 MB PDF)

Figure S11 Analysis of the updated Dunn et al. dataset.
(0.07 MB PDF)

Figure S12 Reanalysis of the Philippe et al. dataset with a reduced taxon sampling.
(0.04 MB PDF)

Figure S13 Reanalysis of the original Schierwater et al. alignment with only the closest outgroup (Choanoflagellata).
(0.04 MB PDF)

Figure S14 Reanalysis of the Philippe et al. dataset with a less complex model.
(0.06 MB PDF)

Table S1 List of errors detected in the dataset of Schierwater et al.
(0.05 MB PDF)

Table S2 List of errors detected in the dataset of Dunn et al.
(0.13 MB PDF)

Text S1 Methods and supporting information.
(0.16 MB DOC)

## Abbreviations

BS: bootstrap support
EST: expressed sequence tag
LBA: long branch attraction
