# Supplementary material for: Resolving Difficult Phylogenetic Questions: Why More Sequences Are Not Enough
Source: PLoS Biol. 2011 Mar 15;9(3):e1000602. doi: 10.1371/journal.pbio.1000602 (PMC3057953; doi:10.1371/journal.pbio.1000602)
Supplement: Figure S2 — Phylogeny of the CDC gene. (0.06 MB PDF) [file pbio.1000602.s004.pdf]

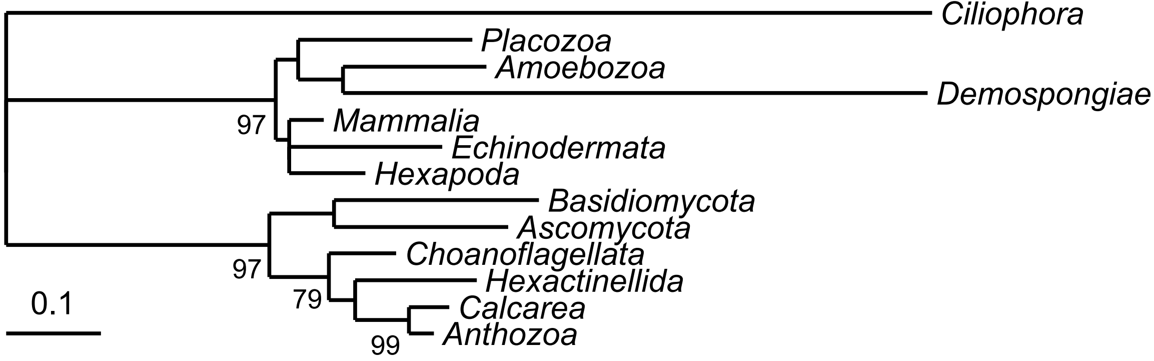

Figure S2: Phylogeny of the CDC gene.

The alignment of Schierwater et al. was used to infer a single gene phylogeny with RAxML (LG+F+Γ model, 100 fast bootstrap replicates). Two different paralogs (cdc42 and rac1) have clearly been mixed. The Ciliophora sequence could represent a third paralog.
