# Supplementary material for: Resolving Difficult Phylogenetic Questions: Why More Sequences Are Not Enough
Source: PLoS Biol. 2011 Mar 15;9(3):e1000602. doi: 10.1371/journal.pbio.1000602 (PMC3057953; doi:10.1371/journal.pbio.1000602)
Supplement: Figure S3 — Phylogeny of the RP3 gene. (0.06 MB PDF) [file pbio.1000602.s005.pdf]

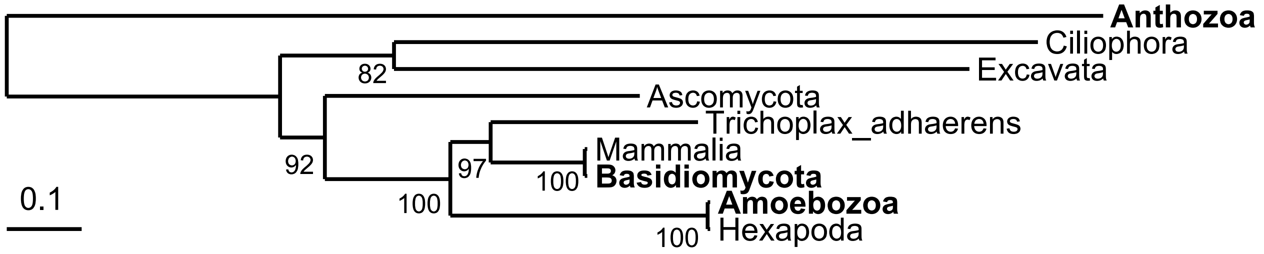

Figure S3: Phylogeny of the RP3 gene.

The alignment of Schierwater et al. was used to infer a single gene phylogeny with RAxML (LG+F+Γ model, 100 fast bootstrap replicates). The Anthozoa sequence belongs to a paralogous family (DNA directed RNA polymerase II). The Basidiomycota sequence is erroneously identical to the mammalian sequence, while the Amoebozoa sequence is actually from an hexapod. All three aberrant sequences are shown in bold.
