# Supplementary material for: Resolving Difficult Phylogenetic Questions: Why More Sequences Are Not Enough
Source: PLoS Biol. 2011 Mar 15;9(3):e1000602. doi: 10.1371/journal.pbio.1000602 (PMC3057953; doi:10.1371/journal.pbio.1000602)
Supplement: Figure S4 — Phylogeny of the EF1 gene. (0.04 MB PDF) [file pbio.1000602.s006.pdf]

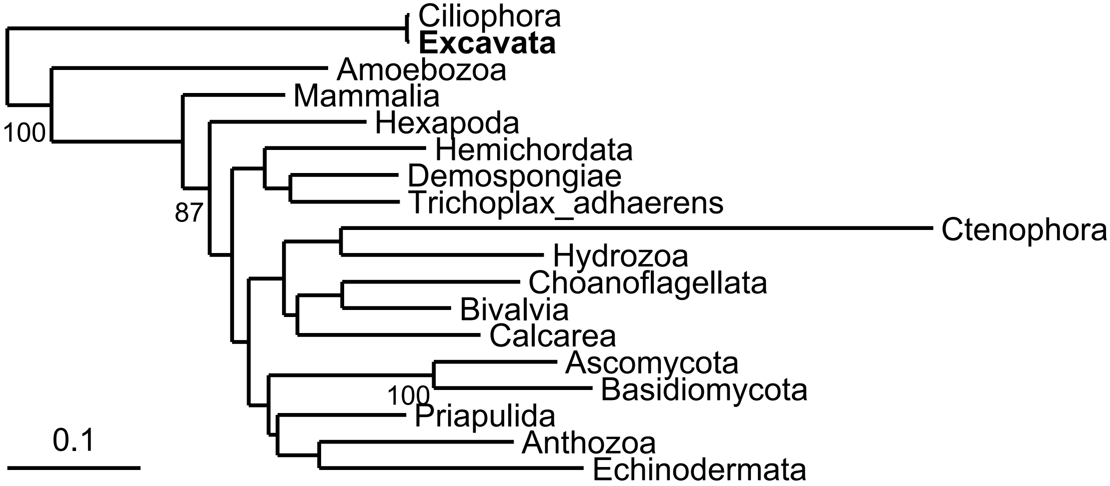


**Figure S4: Phylogeny of the EF1 gene.**

The alignment of Schierwater et al. was used to infer a single gene phylogeny with RAxML (LG+F+$\Gamma$ model, 100 fast bootstrap replicates). The Excavata sequence (in bold) is erroneously identical to the Ciliophora sequence.
