# Supplementary material for: Resolving Difficult Phylogenetic Questions: Why More Sequences Are Not Enough
Source: PLoS Biol. 2011 Mar 15;9(3):e1000602. doi: 10.1371/journal.pbio.1000602 (PMC3057953; doi:10.1371/journal.pbio.1000602)
Supplement: Figure S5 — Phylogeny of the H70 gene. (0.04 MB PDF) [file pbio.1000602.s007.pdf]

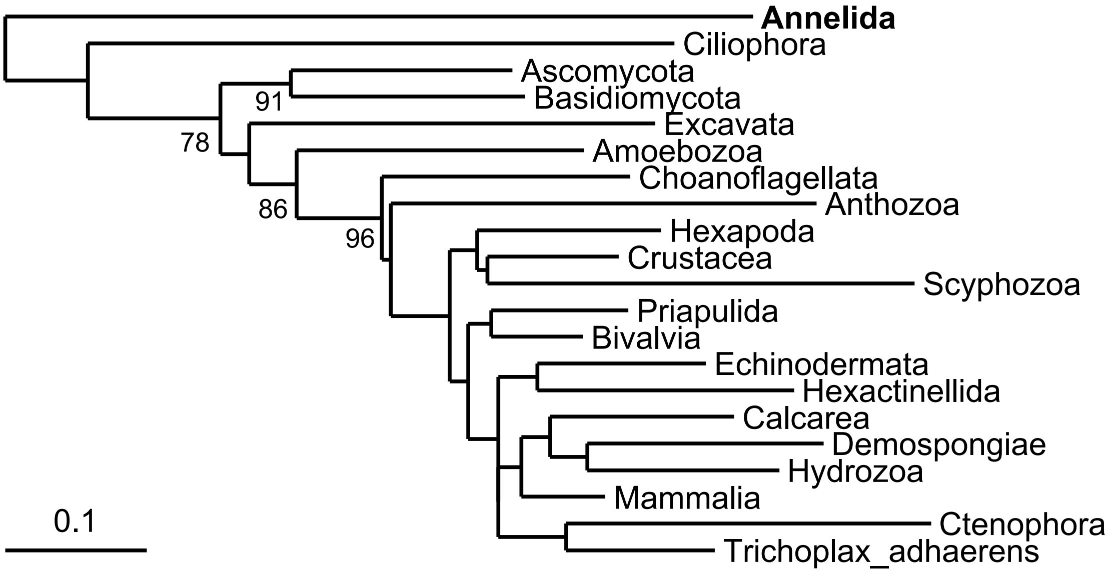

Figure S5: Phylogeny of the H70 gene.

The alignment of Schierwater et al. was used to infer a single gene phylogeny with RAxML (LG+F+Γ model, 100 fast bootstrap replicates). The Annelida sequence (in bold) belongs to a distinct paralogous family, the ER form of hsp70.
