# Supplementary material for: Resolving Difficult Phylogenetic Questions: Why More Sequences Are Not Enough
Source: PLoS Biol. 2011 Mar 15;9(3):e1000602. doi: 10.1371/journal.pbio.1000602 (PMC3057953; doi:10.1371/journal.pbio.1000602)
Supplement: Figure S6 — Phylogeny of the PAX gene. (0.04 MB PDF) [file pbio.1000602.s008.pdf]

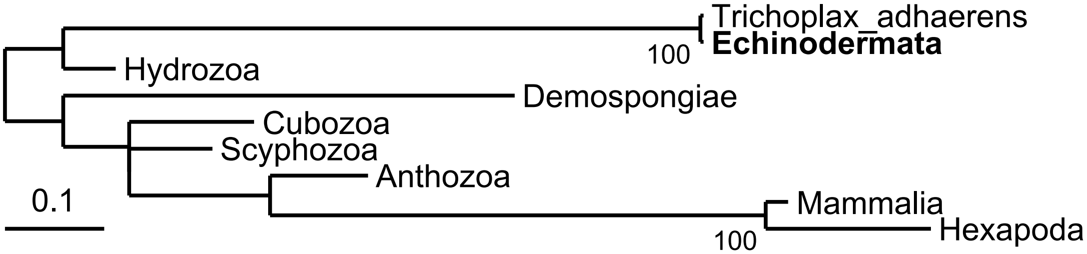

Figure S6: Phylogeny of the PAX gene.

The alignment of Schierwater et al. was used to infer a single gene phylogeny with RAxML (LG+F+Γ model, 100 fast bootstrap replicates). The Echinodermata sequence (in bold) is erroneously identical to the Placozoa sequence. Moreover, orthology is difficult to assess.
