# Supplementary material for: Resolving Difficult Phylogenetic Questions: Why More Sequences Are Not Enough
Source: PLoS Biol. 2011 Mar 15;9(3):e1000602. doi: 10.1371/journal.pbio.1000602 (PMC3057953; doi:10.1371/journal.pbio.1000602)
Supplement: Figure S8 — Phylogeny of the CO2 gene. (0.06 MB PDF) [file pbio.1000602.s010.pdf]

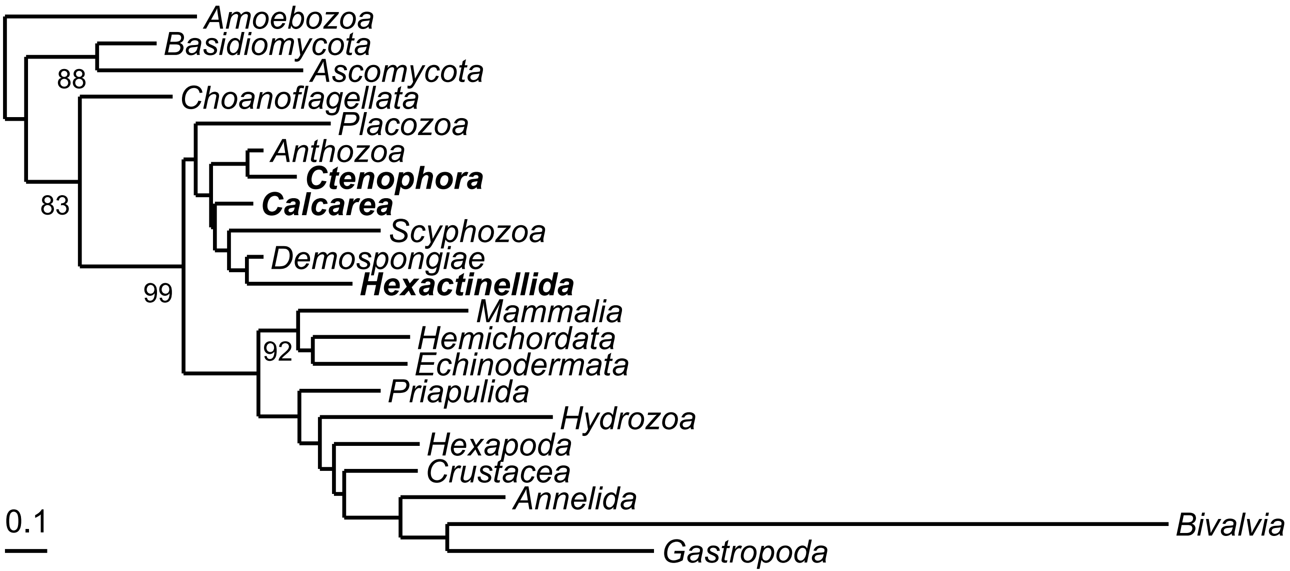

Figure S8: Phylogeny of the CO2 gene.

The alignment of Schierwater et al. was used to infer a single gene phylogeny with RAxML (LG+F+Γ model, 100 fast bootstrap replicates). The Calcarea and Hexactinellida sequences are misidentified and are actually from demosponges (see Fig. S1). Similarly, the Ctenophora sequence is almost identical to the Anthozoa sequence (thus likely also a misidentification). All three sequences are shown in bold.
