# Supplementary material for: Resolving Difficult Phylogenetic Questions: Why More Sequences Are Not Enough
Source: PLoS Biol. 2011 Mar 15;9(3):e1000602. doi: 10.1371/journal.pbio.1000602 (PMC3057953; doi:10.1371/journal.pbio.1000602)
Supplement: Figure S9 — Taxonomic misidentification for mitochondrial proteins of sponges. (0.06 MB PDF) [file pbio.1000602.s011.pdf]

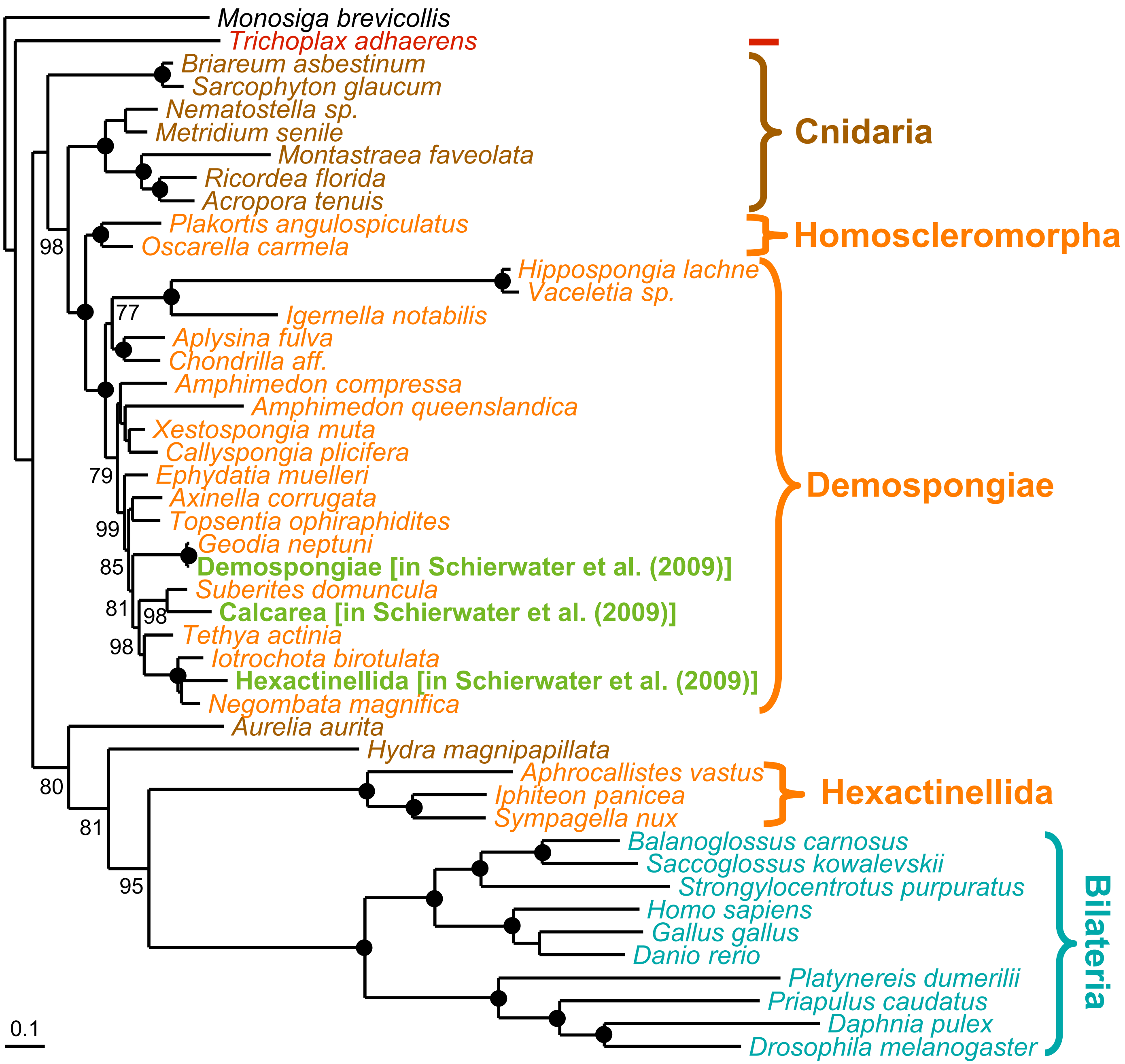

Figure S9: Taxonomic misidentification for mitochondrial proteins of sponges. To verify the taxonomic affiliation of the three poriferan mitochondrial sequences used by Schierwater et al. (indicated in bold), we concatenated 10 well conserved mitochondrial proteins (at6, co1, co2, co3, cyb, nd1, nd2, nd3, nd4, nd5) with a rich taxon sampling, particularly from demosponges and hexactinellids. The resulting alignment (3,136 positions) was analysed with a mtREV+F+Γ model using RAxML. Bootstrap support was computed with 100 replicates and branches supported at 100% are indicated by a bullet. Surprisingly the three poriferan sequences used by Schierwater et al. are deeply nested within Demospongiae. In particular, the hexactinellida sequence does not cluster with the other hexactinellid sequences. It is therefore very likely that the Calcarea and Hexactinellida sequences used by Schierwater et al. are in fact demosponges.
