# Supplementary material for: Resolving Difficult Phylogenetic Questions: Why More Sequences Are Not Enough
Source: PLoS Biol. 2011 Mar 15;9(3):e1000602. doi: 10.1371/journal.pbio.1000602 (PMC3057953; doi:10.1371/journal.pbio.1000602)
Supplement: Figure S10 — Analysis of the revised Schierwater et al. dataset. (0.04 MB PDF) [file pbio.1000602.s012.pdf]

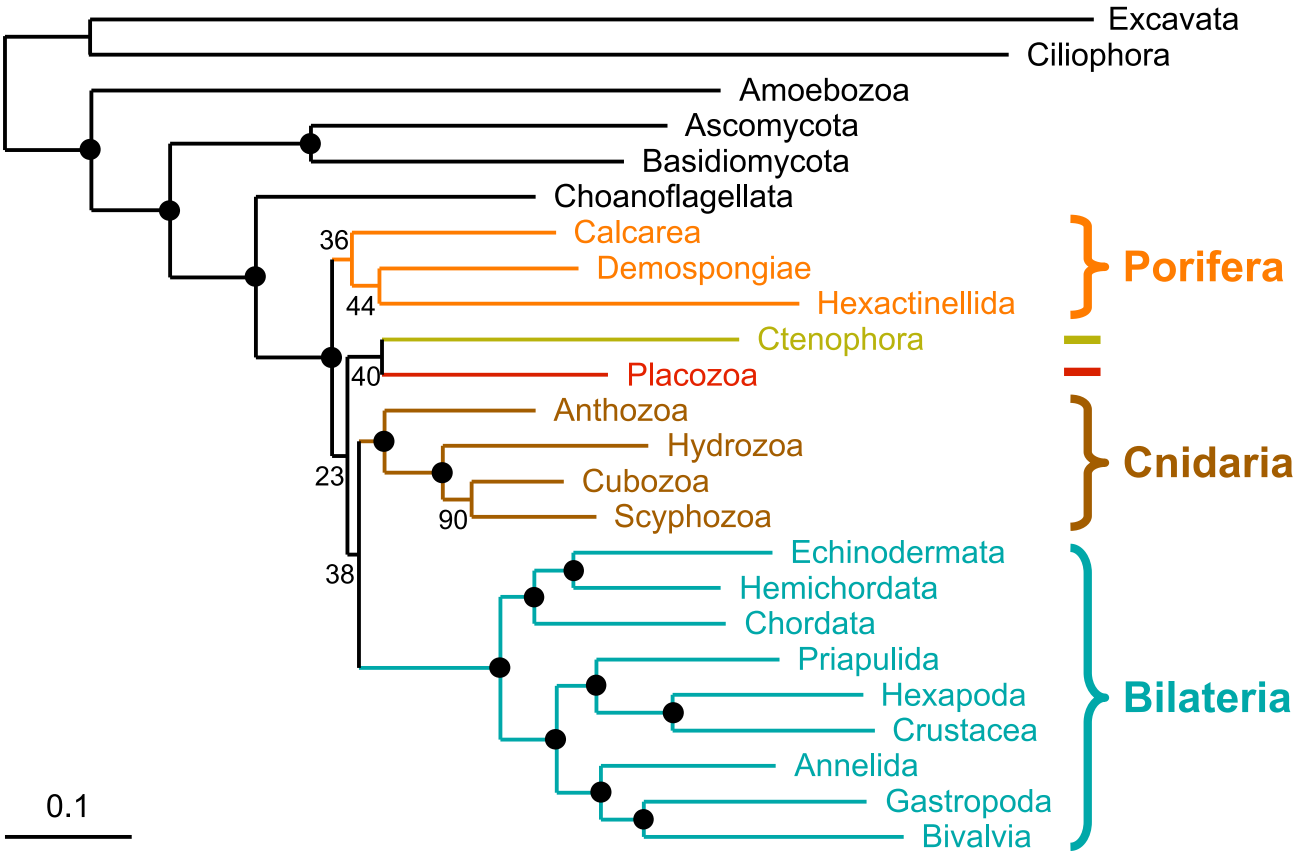

Figure S10: Analysis of the revised Schierwater et al. dataset.

Numerous discrepancies were corrected in the alignments (Table S1) and a few genes were discarded due to dubious orthology assignment; 14,112 unambiguously aligned positions were retained. The tree was inferred as in the original study, i.e., using RAxML with a GTR+Γ model for nucleotide sequences and a LG+F+Γ model for protein sequences.
