# Supplementary material for: Resolving Difficult Phylogenetic Questions: Why More Sequences Are Not Enough
Source: PLoS Biol. 2011 Mar 15;9(3):e1000602. doi: 10.1371/journal.pbio.1000602 (PMC3057953; doi:10.1371/journal.pbio.1000602)
Supplement: Figure S11 — Analysis of the updated Dunn et al. dataset. (0.07 MB PDF) [file pbio.1000602.s013.pdf]

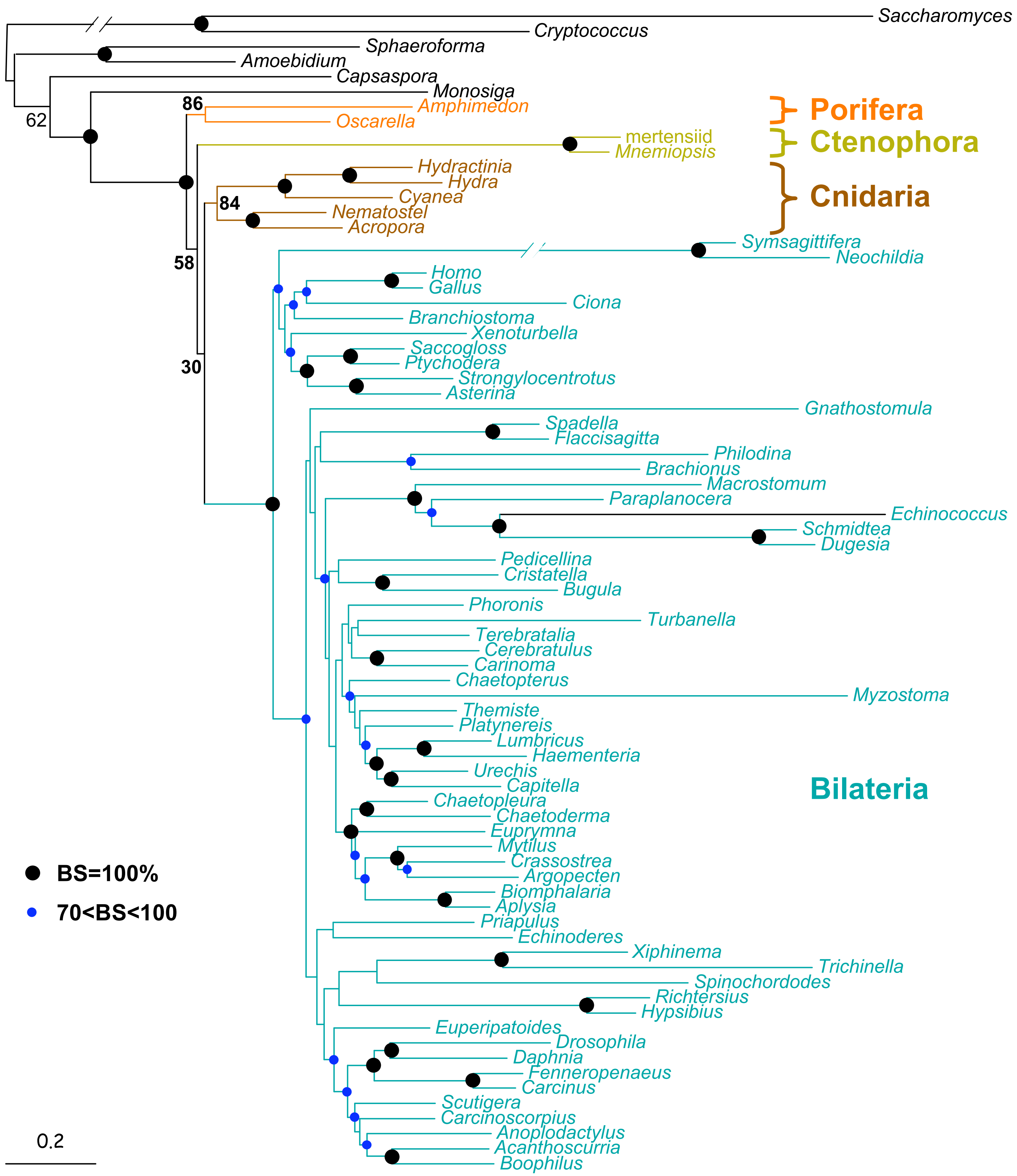

Figure S11: Analysis of the updated Dunn et al. dataset.
Alignments of Dunn et al. (2008) were manually corrected for undisputable errors (see Table S2) and completed with newly available sequences. There are 18,463 positions and only 35.6% of the cells are missing. The tree was inferred with the CAT+Γ model using PhyloBayes (100 bootstrap replicates). Nodes supported at 100% are indicated by black bullets. Within Bilateria, support below 70% is not indicated, while support between 70 and 99% is indicated by blue bullets.
