# Supplementary material for: Resolving Difficult Phylogenetic Questions: Why More Sequences Are Not Enough
Source: PLoS Biol. 2011 Mar 15;9(3):e1000602. doi: 10.1371/journal.pbio.1000602 (PMC3057953; doi:10.1371/journal.pbio.1000602)
Supplement: Figure S12 — Reanalysis of the Philippe et al. dataset with a reduced taxon sampling. (0.04 MB PDF) [file pbio.1000602.s014.pdf]

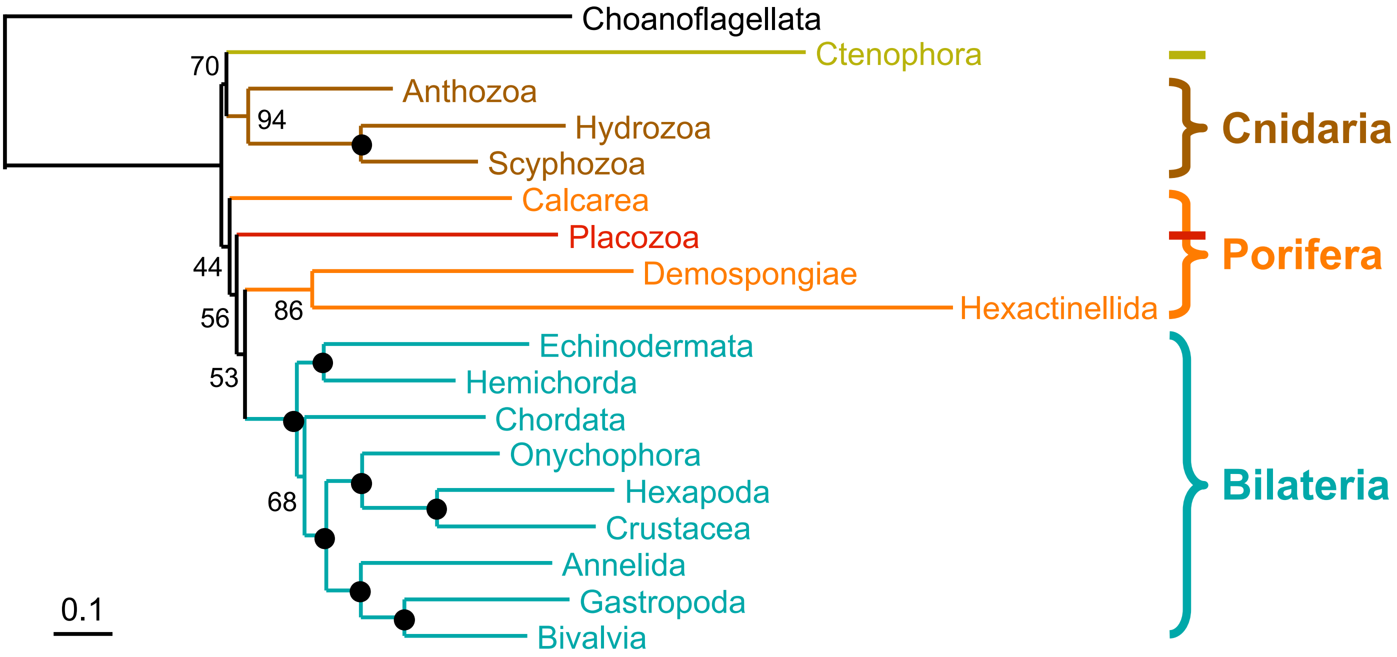

Figure S12: Reanalysis of the Philippe et al. dataset with a reduced taxon sampling.

Both gene selection and inference method (PhyloBayes using the CAT+Γ model) were exactly the same as in the original study. There are 128 genes and 30,257 unambiguously aligned positions.
