# Supplementary material for: Resolving Difficult Phylogenetic Questions: Why More Sequences Are Not Enough
Source: PLoS Biol. 2011 Mar 15;9(3):e1000602. doi: 10.1371/journal.pbio.1000602 (PMC3057953; doi:10.1371/journal.pbio.1000602)
Supplement: Figure S13 — Reanalysis of the original Schierwater et al. alignment with only the closest outgroup (Choanoflagellata). (0.04 MB PDF) [file pbio.1000602.s015.pdf]

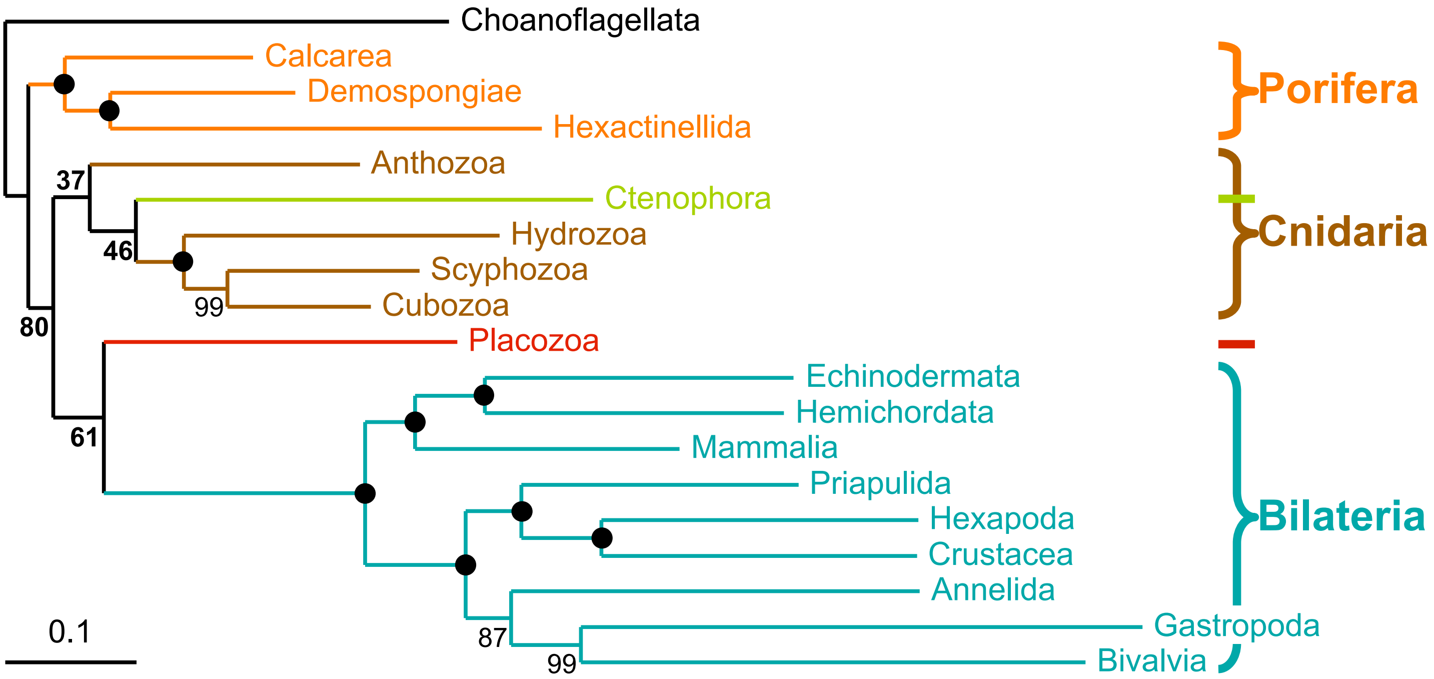

Figure S13: Reanalysis of the original Schierwater et al. alignment with only the closest outgroup (Choanoflagellata).
The 17,101 positions of the original alignment (morphological and structural characters have been excluded) were analysed with the same model as in the original study (i.e., RAxML with a mixed GTR+Γ and LG+F+Γ model, 100 fast bootstrap replicates). Branches supported at 100% are indicated by a bullet.
