# Supplementary material for: Resolving Difficult Phylogenetic Questions: Why More Sequences Are Not Enough
Source: PLoS Biol. 2011 Mar 15;9(3):e1000602. doi: 10.1371/journal.pbio.1000602 (PMC3057953; doi:10.1371/journal.pbio.1000602)
Supplement: Figure S14 — Reanalysis of the Philippe et al. dataset with a less complex model. (0.06 MB PDF) [file pbio.1000602.s016.pdf]

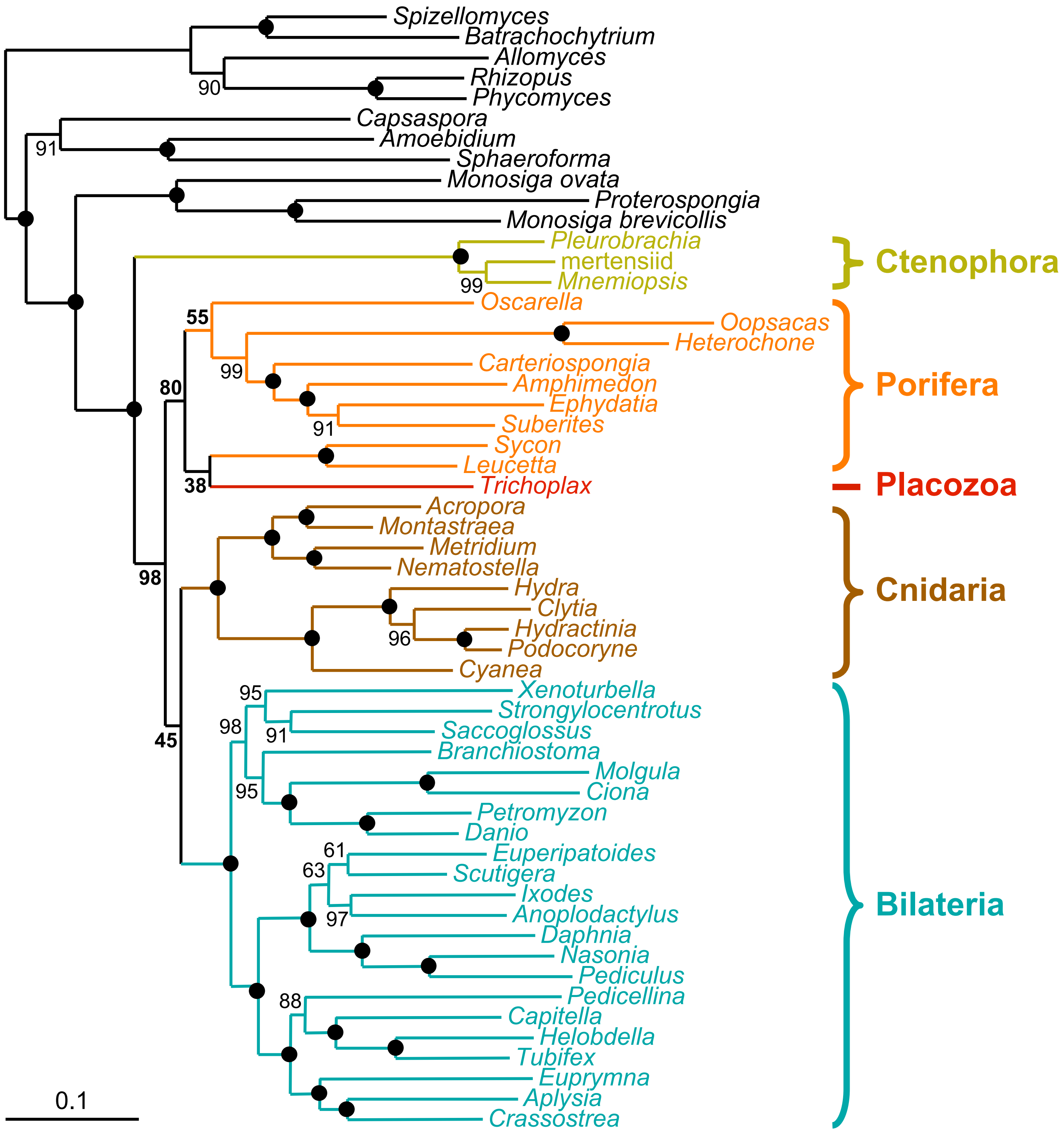

Figure S14: Reanalysis of the Philippe et al. dataset with a less complex model.

The original dataset was reanalysed using RAxML with the WAG+F+Γ model, which has a less good fit than the CAT+Γ model used in the original study.
