# Supplementary material for: Resolving Difficult Phylogenetic Questions: Why More Sequences Are Not Enough
Source: PLoS Biol. 2011 Mar 15;9(3):e1000602. doi: 10.1371/journal.pbio.1000602 (PMC3057953; doi:10.1371/journal.pbio.1000602)
Supplement: Table S1 — List of errors detected in the dataset of Schierwater et al. (0.05 MB PDF) [file pbio.1000602.s017.pdf]

Table S1. List of errors detected in the dataset of Schierwater et al. [1].
Gene names are those of the original study. 'Contamination' includes both biological and *in silico* contaminations, as we cannot determine the origin of reported errors.

Gene	Frameshift	Point mutations	Paralogy	Contamination
bp143				
bp156	Choanoflagellata (70), Hemichordata (45)	Hydrozoa (~10)		
bp169	Choanoflagellata (~15), Chromalveolata (15), Hydrozoa (~10)			
bp174				
bp179	Choanoflagellata (~10), Echinodermata (10)	Hydrozoa (~10)		
bp184	Bivalvia (55), Hydrozoa (40)			
bp186				
bp193				
bp199				
bp200		Choanoflagellata (8)		
bp211				
bp213		Hydrozoa (18)		
bp225				
bp232	Gastropoda (~10)			
bp233				
bp235	Echinodermata (~100)			
bp242				
bp243				
ACT				
BRA				
BTU				
CDC			2 or 3 groups of paralogs	
DCR			very short, some are non homologous	
EF1		Choanoflagelatta (5)		Excavata=Tetrahymena
H70			all sequences are the cytoplasmic HSP70, but Annelida sequence wich is the ER form	
NAK				
PAX			mix of paralogs	Echinodermata=Trichoplax
RAS			2 paralogs (Gastropoda and hydrozoa)	Cubozoa=Insecta
RB2				
RP3			Anthozoa is a paralog	
TF1				
rrna12S				
rrna16S				
rrna18S				
rrna28S				Hexactinellida (several misalignments)
rrna58S			Basidiomycota=non homologous	
AT6			Hemichordata=non homologous	Hexactinellida=Demospongiae, Calcarea=Demospongiae
AT8				
CO1				Hexactinellida=Demospongiae
CO2				Ctenophora=Anthozoa, Hexactinellida=Demospongiae, Calcarea=Demospongiae
CO3		Echinodermata (23)		Hexactinellida=Demospongiae, Calcarea=Demospongiae
CYB				Hexactinellida=Demospongiae
N4L				Hexactinellida=Demospongiae
ND1	Gastropoda	Gastropoda(6)		Hexactinellida=Demospongiae
ND2				Hexactinellida=Demospongiae
ND3				Hexactinellida=Demospongiae
ND4				Hexactinellida=Demospongiae
ND5	Hexactinellida (~30), Trichoplax (~15)	Echinodermata (~20)		Hexactinellida=Demospongiae
ND6				Hexactinellida=Demospongiae
