# Supplementary material for: Resolving Difficult Phylogenetic Questions: Why More Sequences Are Not Enough
Source: PLoS Biol. 2011 Mar 15;9(3):e1000602. doi: 10.1371/journal.pbio.1000602 (PMC3057953; doi:10.1371/journal.pbio.1000602)
Supplement: Table S2 — List of errors detected in the dataset of Dunn et al. (0.13 MB PDF) [file pbio.1000602.s018.pdf]

Table S2. List of errors detected in the dataset of Dunn et al. [2].
Gene names are those of the original study. 'Contamination' includes both biological and *in silico* contaminations, as we cannot determine the origin of reported errors. However, we suspect that the biological material was contaminated as contaminating sequences exclusively belong to parasites. Cases of paralogy are often detected for species not used in the original study, especially for Bilateria, and therefore did not affect the original analysis. Misassignment of paralogs as orthologs is extremely rare and constitutes a negligible issue for this dataset.

gene	frameshift	point mutations	paralogy	contamination/alignment
tribe1006		Branchiostoma(5)		
tribe1007	Acanthosciura(5), Crassostrea (~50)			
tribe1009	Homo(20), Macrostomum(40), Echinococcus(10), Trichinella(10), Oscarella(30)			
tribe1012				
tribe1013	Echinoderes(4, perhaps more)	Branchiostoma(1)		
tribe1015	Macrostomum(90), Euprymna(5)	Boophilus(2)	Heterodera+Meloidogyne(divergent copies, in-paralog)	
tribe1020			Daphnia (out-paralog)	
tribe1026	Crassostrea(50), Euprymna(20)		Myzus (in-paralog)	Scutigera (~50 misaligned), Euprymna (~50 misaligned)
tribe1041	Cristatella(2), Euperipatoides(2), Gallus(20), Paraplanocera(3)			
tribe1046	Eupryma(6?)	Hydra(~5)	Daphnia(3, in-paralog?), Locusta(2, in-paralog)	
tribe1047	Trichinella(20), Carcinus(6), Acanthosciura (10)			
tribe1050		Ptychodera(3)		
tribe1051	Acanthoscurria(3), Gallus(3), Lumbricus(3)	Xiphinema(1)		
tribe1054			Onychiurus(2, in-paralog)	
tribe1077		Turbanella(2)	Drosophila(2, divergent, in-paralog)	
tribe1081	Acanthosciura(25)			
tribe1099	Platynereis(6)			
tribe1118	Carcinus(7), Biomphalaria(50)	Trichinella(6)		
tribe1121	Nematostella (10)	Hydra(1), Carinoma(2), Gnathostomula(2)		
tribe1129	Trichinella(5)			
tribe1132				
tribe1136	Trichinella(20)	Amoebidium (1)		
tribe1162	Biomphalaria(6), Macrostomum(50)			
tribe1165	Brachionus(5)	Anoplodactylus(3), Asterina(1)		
tribe1169	Ptychodera(20), Trichinella(60), Xiphinema(3)			
tribe1170	Biomphalaria(10), Acanthosciura(10)	Branchiostoma(1), Brachionus(1)		
tribe1181	Crassostrea(10)			
tribe1187		Branchiostoma(~10), Hydra(7)		
tribe1190		Trichinella(2), Carinoma(1)		
tribe1200				
tribe1204				
tribe1244	Trichinella(intron), Fenneropenaeus(20), Ciona(40)			
tribe1245			Hirudo, within Lepipdotera (in-paralog) + 3 divergent sequences (Nematostella, Strongylocentrotus, Laupala, likely, in-paralog)	
tribe1292	Gallus(10), Macrostomum(20)		Monosiga (~60 misaligned)	
tribe1294	Richtersius(30), Branchiostoma(10)			
tribe1335				
tribe1374	Biomphalaria(10)			Capitella (~20 misaligned), Lumbricus (~40 misaligned), Euprymna (~30 misaligned)
tribe1378		Trichinella(2)		
tribe1381	Gnathostomula(10)	Hydra(2)		

gene	frameshift	point mutations	paralogy	contamination/alignment
tribe1409	Biomphalaria(10)			
tribe143	Schmidtea(5)	Acropora(1), Argopecten(2), Crassostrea(2), Hypsibius(1)		Symsagitiferra (Chlorophyta)
tribe1489		Philodina(1)		
tribe156	Bugula(10), Carcinoscorpius(4), Monosiga(70), Saccoglossus(55)	Brachionus(2), Cristatella(1), Hydractinia(12), Oscarella(6), Paraplanocera(4), Platynereis(1)		
tribe1646	Macrostomum(10)			
tribe169	Brachionus(30), Branchiostoma(8), Chaetopleura(10), Echinococcus(50), Euprymna(30), Hydractinia(7), Mnemiopsis(6), Monosiga(20)			Biomphalaria (~50 misaligned)
tribe174	Asterina(30), Carcinoscorpius(15), Euperipatoides(10), Phoronis(80)	Acanthoscurria (20), Biomphalaria(4), Cristatella(2), Echinoderes(2)		
tribe179	Monosiga(8), Oscarella(10), Trichinella(10), Macrostomum(80), Strongylocentrotus(6), Scutigera(10)	Trichinella(3), Spadella(1), Crassostrea(1), Carinoma(1), Hypsibius(2), Hydractinia(5)		Neochildia (Microsporidia)
tribe184	Acropora(12),Biomphalaria(40), Carcinoscorpius(3), Chaetopleura(3), Echinococcus(5), Euprymna(30), Hydractinia(30), Macrostomum(20), Mytilus(60), Oscarella(10), Symsagitifera(7), Trichinella(75)	Brachionus(2), Xiphinema(10)		
tribe186	Amoebidium(30), Capsaspora(40), Oscarella(47)	Anoplodactylus(2), Hydractinia(1), Mnemiopsis(2), Monosiga(3), Turbanella(5)		Carcinoscorpius (~20 misaligned)
tribe193	Spadella(8)	Argopecten(1), Echinococcus(5), Homo(3)		
tribe199	Dugesia(20)	Boophilus(2), Chaetopterus(1), Eyprymna(2)		Neochildia (Microsporidia)
tribe200	Acanthoscurria(5), Acropora(10), Capitella(60), Fenneropenenaeus(5), Macrostomum(~10)	Chaetopleura(3), Hydractinia(1), Monosiga(5)		Cristatella (~70 misaligned)
tribe211	Biomphalaria(30)	Chaetopleura(2), Ptychodera(2), Spadella(2)		Symsagitiferra (Chlorophyta)
tribe213		Euprymna(1), Monosiga(3),Sphaeroform a(2), Trichinella(13)		Symsagitiferra (Chlorophyta, ~40 misaligned)
tribe225	Biomphalaria(30), Branchiostoma(10), Echinococcus(10), Sphaeroforma(10)	Spadella(1), Trichinella(1)		
tribe226	Euprymna(7)	Cerebratulus(2)		
tribe232	Acropora(8), Amoebidium(2), Aplysia(14), Capitella(10), Capsaspora(6), Fenneropeneaus(13), Gallus(5), Myzostoma(15), Strongylocentrotus(6)	Cyanea(3), Macrostomum(5)		Trichinella (Ascaris)
tribe233	Anoplodactylus(intron), Capitella(7)			
tribe235	Boophilus(10), Ciona(intron), Gallus(20), Strongylocentrotus(10)			Neochildia (Microsporidia)
tribe236	Fenneropenenaeus(25)	Biomphalaria(1)		
tribe241	Aplysia(5), Capsaspora(4), Mytilus(18)			
tribe242	Argopecten(12), Biomphalaria(4), Crassostrea(20)			
tribe243	Cyanea(5), Scutigera(3)	Anoplodactylus(2), Echinoderes(2)		Macrostomum(choano?), Symsagitiferra (Chlorophyta)

gene	frameshift	point mutations	paralogy	contamination/alignment
tribe260	Acropora(3), Haementeria(4), Phoronis(7), Trichinella(30), Schmidtea(12), Strongylocentrotus(5)	Branchiostoma(3)		
tribe263	Crassostrea(120), Fenneropenaeus(8), Euprymna(15), Capitella(6)	Saccoglossus(10, bad genetic code), Echinococcus(6), Paraplanocera(12), Dugesia(5), Asterina(11)		Hydra(Artemia)
tribe268				
tribe271				
tribe272		Acropora(2), Capitella(20), Echinococcus(1), Pedicellina(1), Platynereis(4)		
tribe273	Monosiga(10)	Trichinella(1), Turbanella(2)		Priapulus (Phoronis)
tribe279		Acanthoscurria(10), Cryptococcus(6)		Neochildia (Microsporidia)
tribe281	Asterina(5), Trichinella(5)	Oscarella(2), Schmidtea(1)	Tribolium (2)	
tribe287	Argopecten(17), Ciona(5)	Monosiga(1), Paraplanocera(1)		Symsagitiferra (Ciliates)
tribe288	Dugesia(3), Strongylocentrotus(25), Themiste(intron)	Capsaspora(52)		Symsagitiferra (Ancyromonas)
tribe299	Chaetopleura(7)	Anoplodactylus(1), Biomphalaria(1), Euperipatoides(1), Gnathostomula(2), Xiphinema(3)		Symsagitiferra (Chlorophyta?)
tribe305	Daphnia(20)	Brachionus(2), Chaetopleura(2), Cyanea(1), Echinococcus(1), Scutigera(3)		Symsagitiferra (Ciliates)
tribe319	Anoplodactylus(6)	Euperipatoides(1), Hydractinia(5)		
tribe320	Anoplodactylus(2)	Crassostrea(1)		Symsagitiferra (Cnidaria?)
tribe321		Chaetopleura(2)		Acanthoscurria (Triticum)
tribe325	Biomphalaria(13), Haementeria(50), Macrostomum(10)	Aplysia(4)		Branchiostoma (not clear donnor)
tribe330	Argopecten(4), Crassostrea(40), Fenneropenaeus(4)	Strongylocentrotus (24), Ptychodera(3), Cyanea(7), Daphnia(2), Paraplanocera(5), Schmidtea(6)		Dugesia (Gallus), Xiphinema (Lumbricus), Asterina (~60 misaligned), Cyanea (~60 misaligned), Nematostella (~40 misaligned)
tribe333	Flaccisagitta(30), Capsaspora(10), Euprymna(10)	Biomphalaria(2)	Paracentrotus+Strongylocentrotus (3, in-paralog), Saccoglossus(3) and several others (drosophila, etc.)	
tribe340	Aplysia(10)			
tribe345	Acropora(12), Ciona(5), Dugesia(21)			
tribe351	Euperipatoides(5), Hydractinia(10)	Turbanella(1)		Priapulus (Alveolata)
tribe352	Biomphalaria(25), Crassostrea(4), Daphnia(5), Echinococcus(7)	Sphaeroforma(20), Spinochordodes(10)		
tribe355		Argopecten(2), Branchiostoma(2), Euperipatoides(2)		
tribe356	Brachionus(15), Chaetoderma(15),Mytilus(4)	Chaetopleura(2), Echinoderes(1), Gnathostomula(1), Trichinella(3), Turbanella(1)		Symsagitiferra (Amoebozoa?)

gene	frameshift	point mutations	paralogy	contamination/alignment
tribe368	Aplysia(10), Mytilus(60), Saccoglossus(5)	Saccoglossus(17, bad genetic code), Ciona(82), Schmidtea(7), Euperipatoides(7)		Ptychodera (~200 misaligned)
tribe369		Fenneropenaeus(3), Asterina(21)		Hydra (Artemia), Saccoglossus (Mus)
tribe380	Lumbricus(10), Chaetopterus(5)	Saccoglossus(10, bad genetic code), Ciona(62), Paraplanocera(16)		
tribe400	Flaccisagitta (25), Schmidtea(110)	Fenneropenaeus(4), Asterina(34, bad code), Ptychodera(5), Dugesia(9), Boophilus(3)		Saccoglossus (Mus), Dugesia (Gallus)
tribe435	Scutigera(3), Xiphinema(30)	Anoplodactylus(1), Xiphinema(~5)		
tribe460	Eupryma(40)	Chaetopterus(1), Eupryma(30), Trichinella(2), Richtersius(3)		Strongylocentrotus (chimerical gene, the first 40 amino acids are from Rho guanine nucleotide exchange factor 3)
tribe495	Carcinus(5), Trichinella(20), Biomphalaria(3)	Euprymna(1), Monosiga(3), Schmidtea(1), Amoebidium(5)		
tribe507	Biomphalaria(10), Mytilus(6)	Daphnia(1), Turbanella(2)		
tribe508		Trichinella(3), Xiphinema(1), Richtersius(1)		
tribe532	Oscarella(4)			
tribe542	Cerebratulus (~10)	Bugula(1)	Schmidtea(divergent, in-paralog)	
tribe550			several paralogs (without clear structure)	
tribe572	Capsaspora(8), Branchiostoma (6), Aplysia(10), M. ovata(10)	Branchiostoma(2)	Rhodnius, Hymenoptera(divergent, in-paralog?), Brugia (in-paralog), Tribolium (divergent, in-paralog?)	
tribe585	Macrostomum(5), Gallus (~30)	Hypsibius(7)	duplication at the base of Hexapoda	
tribe586			Caenorhabditis (divergent, in-paralog?)	
tribe593	Capitella(10), Chaetopterus(intron)			
tribe613	Ferrenopenaeus(3)		Apis	
tribe619	Ferrenopenaeus(10)	Acropora(1)		
tribe622	Biomphalaria(20)			Trichinella (Pseudomonas)
tribe629	Echinococcus(3), Strongylocentrotus(3), Mytilus(3)	Gnathostomula(1), Branchiostoma(10)		
tribe632			numerous divergent copies in Hexapoda and Nematoda	
tribe646	Spinochordodes(6), Biomphalaria(3)	Acanthoscurria(1)		
tribe647	Macrostomum(2)	Branchiostoma(10)		
tribe683	Turbanella(10)	Mnemiopsis(3)	Ixodes (3, in-paralog), Saccoglossus(3), Folsomia(2), Lumbricus(2), several hexapoda, many others	Symsagittifera (Cyanobacteria)
tribe686			many species	
tribe696	Trichinella(5)	Strongylocentrotus(1)		
tribe700	Oscarella(40)		bombyx (2, divergent, in-paralog?)	
tribe703	Crassostrea(50), Echinococcus(10), Gallus(~10), Lumbricus(4), Macrostomum(~25), Symsagitifera(11), Trichinella(10)	Hydra(1), Shaeroforma(2)		
tribe716	Hydra(10), Euprymna(10)			
tribe717	Daphnia(7)	Hydractinia(4)	Drosophila(2)	Acanthoscurria (angiosperm, + frameshift of 68 aa)
tribe726			Brugia(2), Mammalia(2), mosquitoes(2)	
tribe739	Ferrenopenaeus(4)	Euperipatoides(1)	Myzus(2), Drosophila(2)	
tribe740	Euprymna(5)	Mytilus(1)		
tribe742		Asterina(1), Crassostrea(2)	Tribolium(2), Vertebrata(2), Platyhelminthes(2), Symsagittifera(2)	
tribe756		Monosiga(3)	Helobdella(2), Drosophila(2)	
tribe764	Aplysia(6)		Coleoptera(2)	
tribe765	Trichinella(6)		Capitella(2)	
tribe766				Carcinoscorpius (~10 misaligned), Carcinus (~10 misaligned)

gene	frameshift	point mutations	paralogy	contamination/alignment
tribe768	Homo(5)	Branchiostoma(2)		
tribe782	Gallus(5), Biomphalaria(10), Euperipatoides(4)			
tribe783	Chaetopleura(3)	Anoplodactylus(2)		
tribe793	Crassostrea(2), Biomphalaria(40)	Mnemiopsis(1)		
tribe801	Argopecten(10)		Nematostella(2, divergent), Insecta(several)	
tribe805	Drosophila(2), Echinococcus(5)	Crassostrea(2), Daphnia(1), Oscarella(3)		
tribe831				
tribe832	Macrostomum(20),Xenoturbella(4), Euprymna(20),Fenneropenaeus(20)	Themiste(2), Mytilus(2)	Drosophila(2, divergent)	
tribe833	Branchiostoma(15), Cryptococcus(8), Hypsibius(30), Mnemiopsis(3), Trichinella(5)	Acanthoscurria(1)		
tribe850	Dugesia(2), Boophilus(20)	Amoebidium (7)		
tribe852	Symsagittifera(4)	Branchiostoma(1)		
tribe858		Branchiostoma(2), Schmidtea(1)	many many paralogs	Asterina (Bacteria)
tribe861	Cristatella(4), Schmidtea(2)			
tribe868				
tribe893			Monosiga(2)	
tribe895	Branchiostoma(3), Biomphalaria(70)			
tribe896	Xiphinema(10), Lumbricus(3)			Symsagitiferra (Chlorophyta)
tribe905		Echinococcus(1)	Caenorhabditis(2,in), Prisrionchus(2,in), Drosophila(divergent)	
tribe906		Crassostrea(5)	Drosophila(2, divergent), Mammalia(2)	
tribe916		Echinococcus(1)	Lepidoptera(2)	Symsagitiferra (Prokaryota), Monosiga (Rhizopus)
tribe927	Aplysia(3)	Branchiostoma(2)		
tribe930	Eupryma(3)	Biomphalaria(1)	Myzus(2)	
tribe942		Boophilus(50), Branchiostoma(1)	Drosophila(2, divergent)	Oscarella (Pseudomonas)
tribe961	Hydractinia(6)	Cristatella(2),Crassostrea(1), Aplysia(5), Gnathostomula(2)	Helobdella(2, divergent)	
tribe965				
