# Supplementary material for: Resolving Difficult Phylogenetic Questions: Why More Sequences Are Not Enough
Source: PLoS Biol. 2011 Mar 15;9(3):e1000602. doi: 10.1371/journal.pbio.1000602 (PMC3057953; doi:10.1371/journal.pbio.1000602)
Supplement: Text S1 — Methods and supporting information. (0.16 MB DOC) [file pbio.1000602.s019.doc]

**Text S1. Methods and Supporting Information**

**Manual checking of alignments**

Whenever possible, sequences in individual gene alignments from studies of Schierwater et al. [1] and Dunn et al. [2] were imported into the taxon-rich gene alignments maintained in the Philippe lab using sequences produced in other labs [2-12]. Otherwise, their alignments were first enriched with all homologs available in GenPept as detected by blast (different E-values were used depending on the specificity of each gene). Then homologous sequences downloaded from the Trace Archive (<http://www.ncbi.nlm.nih.gov/Traces/>) and the EST Database (<http://www.ncbi.nlm.nih.gov/dbEST/>) of GenBank at the National Center for Biotechnology Information (<http://www.ncbi.nlm.nih.gov/>) were added using the program forty (Denis Baurain, personal communication), which is based on blast searches and cap3 assemblies, as well as new features of the program ed from the MUST package [13].

Most of the analyses were carried out manually with the sequence editor ed [13]. Briefly, each sequence from [1,2] was visually compared with its most similar counterparts in order to detect frameshifts (leading to stretches of amino acids highly different from the consensus) and with all its homologs to detect local sequencing errors (visible as unmatched amino acids at highly conserved positions). Many frameshifts and local sequencing errors were confirmed by comparison with sequences made available since the assembly of the datasets used in [1,2]. A brief description of the errors identified is provided in Tables S1 and S2.

To detect taxonomic misidentifications, each individual sequence was blasted against all sequences of the same alignment. Most often, the best hit corresponded to a species known to be closely related to the species of the query sequence; if so no further analysis was performed. Otherwise, an additional blast search was carried out at the NCBI website against the nr database. Such an approach allowed us to confirm that the hemichordate *atp6* gene used in [1] is actually not homologous to other *atp6* genes (see Figure S1). For all suspicious cases (i.e., putative contamination or hidden paralogy), a phylogenetic tree was inferred with RAxML [14] using a LG+F+Γ model. This applies to eight genes from the Schierwater et al. dataset, here re-analysed with their original taxon sampling (Figures S1-S8). To confirm the twofold misidentification of calcarean and hexactinellid sponge mitochondria, ten mitochondrial genes were concatenated using Scafos [15] and analysed with a denser taxon sampling using a mtREV+F+Γ model (Figure S9).

To automatically check the orthology of each gene, we applied the same protocol as in [4], except that trees were inferred with RAxML [14] using a LG+F+Γ model. Briefly, this protocol evaluates whether every statistically supported branch in a single gene phylogeny is congruent with the concatenated phylogeny (Denis Baurain, unpublished). Since incomplete sequences may display very unstable positions in inferred trees, sequences with more than 50% missing characters were discarded. Consequently, errors affecting partial sequences cannot be detected with this approach. Moreover, while undisputable, some incongruent cases may fail to achieve significance at a bootstrap support (BS) threshold of 70%. For instance, in the *cox2* alignment used by Schierwater et al. (Figure S8), Ctenophora are oddly similar to Anthozoa, though this grouping does not receive a high BS. Finally, single gene trees may be congruent with the concatenated topology because the species set considered is too small. Hence, demosponges and hexactinellids being true sister groups, the aforementioned mitochondrial hexactinellid contamination can only be unveiled with a dense taxon sampling, in which this supposedly hexactinellid sequence emerges from within demosponges (Figure S9). Similarly, the “excavate” *ef1* gene from Schierwater et al. is correctly located as a sister group of Ciliophora (given the scarce taxon sampling), but should certainly not be identical to the latter sequence (Figure S4).

For every gene used by Schierwater et al. [1] and Dunn et al. [2], we selected unambiguously aligned positions with the program GBlocks [16] using the same stringency criteria as in [4,11,17-21]. Finally, we assembled the updated concatenated alignments using Scafos [15]. Our corrected datasets contained less amino acid positions than those from original studies: 14,112 versus 17,422 [1] (note that the morphological and structural characters were discarded after verifying that their removal had no impact on the inferred phylogeny nor on its support) and 18,463 versus 21,152 [2]. For Schierwater et al., five genes (*atp8*, *dcr*, *nd4l*, *nd6*, *pax*) were excluded because too few positions were retained after filtering or because orthology could not be reliably established. However, since the filtering criteria used in the original study were not provided, it was difficult to investigate the observed difference. In contrast, the criteria used by Dunn et al. for GBlocks (b2=65%, b3=10, b4=5, b5=all) were less stringent than ours (b2=75%, b3=5, b4=5, b5=half), thus explaining the difference. By being more stringent than in the original studies, we have probably excluded the most fast-evolving positions, which led to less saturated datasets. Nevertheless, our use of the same set of criteria for all datasets makes the comparison of their saturation level (Figure 5) particularly fair.

Complete trees obtained from the revised datasets of Schierwater et al. [1] (see also Figure 2B) and Dunn et al. [2] are given as Figures S10 and S11, respectively.

**Importance of outgroup and model selection**

Increasing the number of species is largely recognized as a major avenue to improve the accuracy when investigating difficult phylogenetic questions [22-24]. This is for instance demonstrated in Figure S12, which is discussed in the main text (as Figure 3B). However, the nature of the taxon sampling can be more important than the number of species *per se*. In the case of basal animal relationships, beside issues of ingroup short internal and long terminal branches discussed in the main text, the outgroup plays a prominent role. In particular, a distant outgroup can readily — but artefactually — attract any fast-evolving ingroup lineage [25], such as Ctenophora with nuclear genes or Bilateria with mitochondrial genes. As recently shown for the Philippe et al. dataset [4], the use of the closest possible outgroup seems to be helpful in accurately inferring animal relationships. Here, we demonstrate that the same conclusion holds for the datasets of Schierwater et al. [1] and Dunn et al. [2].

In the case of Schierwater et al., very distantly related and fast-evolving outgroups, such as Excavata and Chromalveolata, were considered, thus potentially exacerbating the long branch attraction (LBA) artefact. To check this possibility, we re-analysed their dataset with the same method as in the original study [1], yet after discarding all but the closest outgroup (Choanoflagellata). The resulting tree, which was inferred from a dataset that had not yet been corrected for the errors reported in Table S1, is nonetheless seriously different (Figure S13) from the published tree [1]. Bilateria no longer emerge at the base of animals (BS=100% in the original study), with this position now occupied by sponges (BS=80%), and Placozoa are now sister-group to Bilateria (BS=61%). In other words, merely using a close outgroup is enough to make the analysis of the Schierwater et al. dataset congruent with the topology of Philippe et al. [4]. It is noteworthy that the sensitivity to LBA is probably enhanced by the inclusion of a large share of mitochondrial genes, for which Bilateria are extremely fast-evolving — and thus more readily attracted by the distant outgroups used.

In the case of Dunn et al., only opisthokonts were used as outgroups, which is definitively better. Unfortunately, the closest outgroup was the choanoflagellate *Monosiga ovata*, which was largely incomplete in the original study (50.5% of missing data). Thus, for half of the positions, the closest outgroup was at best Ichthyosporea, which are not only more distantly related (see Figure S11) but also incomplete (*Capsaspora*, 43.6%, *Sphaeroforma*, 47.9% and *Amoebidium*, 70.1%). This profusion of missing data entails that the operational outgroup was the much more distant fungi. Building on new sequence data, in particular the genome of *Monosiga brevicollis*, we filled many of the holes found in Dunn et al. alignments while checking for other errors (Table S2). This allowed a drastic reduction in the amount of missing data in their alignments (*Monosiga*, 5%, *Capsaspora*, 12.4%, *Sphaeroforma*, 39.3% and *Amoebidium*, 59.3%). The concatenation of these new alignments was then analysed with the CAT+Γ model using PhyloBayes [26] and the robustness assessed through 100 bootstrap replicates. As expected, the completion of outgroup sequences had a major impact on the resulting phylogeny (Figure S11): the fast-evolving Ctenophora no longer emerge at the base of the animals with strong BS [2]; instead sponges emerge first, though with a limited BS (58%). Exactly as with the dataset of Schierwater et al., using a complete close outgroup is enough to make the analysis of the dataset of Dunn et al. congruent with the topology of Philippe et al. [4].

These two experiments (Figures S11 and S13) demonstrate that great care should be taken in the assembly of the outgroup sequences when analysing a difficult phylogenetic question. Importantly, the taxon sampling of the metazoan outgroup has not yet been the subject of a major sequencing effort, so that five of the three Ichthyosporea and of three Choanoflagellata presently available are incomplete (except *Monosiga ovata*). Therefore, to accurately reconstruct the multiple substitutions obfuscating the ancestral sequence at the base of the animal branch, it is needed to deeply sample the outgroup. With the currently scarce taxon sampling, the characterization of the best outgroup is difficult. For instance, is it preferable to use a paraphyletic (i.e., Ichthyosporea + Choanoflagellata) or monophyletic (i.e., Choanoflagellata) outgroup? The two experiments reported above suggest that the presence of distant outgroups (excavates, amoebozoans and also fungi) is detrimental, but refined analyses are needed to settle the question. At any rate, it is highly recommended to analyse datasets with and without outgroups to check the stability of the inferred phylogeny with regard to one of the most disturbing factors.

As discussed in the main text (Figure 4B), using a less complex model of sequence evolution (here, WAG) can seriously enhance non-phylogenetic signal (Figure S14).

**References**

1. Schierwater B, Eitel M, Jakob W, Osigus HJ, Hadrys H, et al. (2009) Concatenated analysis sheds light on early metazoan evolution and fuels a modern "urmetazoon" hypothesis. PLoS Biol 7: e20.

2. Dunn CW, Hejnol A, Matus DQ, Pang K, Browne WE, et al. (2008) Broad phylogenomic sampling improves resolution of the animal tree of life. Nature.

3. Hejnol A, Obst M, Stamatakis A, Ott M, Rouse GW, et al. (2009) Assessing the root of bilaterian animals with scalable phylogenomic methods. Proc Biol Sci.

4. Philippe H, Derelle R, Lopez P, Pick K, Borchiellini C, et al. (2009) Phylogenomics revives traditional views on deep animal relationships. Curr Biol 19: 706-712.

5. Bourlat SJ, Juliusdottir T, Lowe CJ, Freeman R, Aronowicz J, et al. (2006) Deuterostome phylogeny reveals monophyletic chordates and the new phylum Xenoturbellida. Nature 444: 85-88.

6. Matus DQ, Copley RR, Dunn CW, Hejnol A, Eccleston H, et al. (2006) Broad taxon and gene sampling indicate that chaetognaths are protostomes. Curr Biol 16: R575-576.

7. Hausdorf B, Helmkampf M, Meyer A, Witek A, Herlyn H, et al. (2007) Spiralian phylogenomics supports the resurrection of Bryozoa comprising Ectoprocta and Entoprocta. Mol Biol Evol 24: 2723-2729.

8. Witek A, Herlyn H, Ebersberger I, Mark Welch DB, Hankeln T (2009) Support for the monophyletic origin of Gnathifera from phylogenomics. Mol Phylogenet Evol.

9. Witek A, Herlyn H, Meyer A, Boell L, Bucher G, et al. (2008) EST based phylogenomics of Syndermata questions monophyly of Eurotatoria. BMC Evol Biol 8: 345.

10. Bleidorn C, Podsiadlowski L, Zhong M, Eeckhaut I, Hartmann S, et al. (2009) On the phylogenetic position of Myzostomida: can 77 genes get it wrong? BMC Evol Biol 9: 150.

11. Philippe H, Brinkmann H, Martinez P, Riutort M, Baguna J (2007) Acoel flatworms are not platyhelminthes: evidence from phylogenomics. PLoS ONE 2: e717.

12. Marletaz F, Martin E, Perez Y, Papillon D, Caubit X, et al. (2006) Chaetognath phylogenomics: a protostome with deuterostome-like development. Curr Biol 16: R577-578.

13. Philippe H (1993) MUST, a computer package of Management Utilities for Sequences and Trees. Nucleic Acids Res 21: 5264-5272.

14. Stamatakis A, Ludwig T, Meier H (2005) RAxML-III: a fast program for maximum likelihood-based inference of large phylogenetic trees. Bioinformatics 21: 456-463.

15. Roure B, Rodriguez-Ezpeleta N, Philippe H (2007) SCaFoS: a tool for Selection, Concatenation and Fusion of Sequences for phylogenomics. BMC Evol Biol 7 Suppl 1: S2.

16. Castresana J (2000) Selection of conserved blocks from multiple alignments for their use in phylogenetic analysis. Mol Biol Evol 17: 540-552.

17. Philippe H, Lartillot N, Brinkmann H (2005) Multigene analyses of bilaterian animals corroborate the monophyly of Ecdysozoa, Lophotrochozoa, and Protostomia. Mol Biol Evol 22: 1246-1253.

18. Baurain D, Brinkmann H, Philippe H (2007) Lack of resolution in the animal phylogeny: closely spaced cladogeneses or undetected systematic errors? Mol Biol Evol 24: 6-9.

19. Jimenez-Guri E, Philippe H, Okamura B, Holland PW (2007) Buddenbrockia is a cnidarian worm. Science 317: 116-118.

20. Delsuc F, Tsagkogeorga G, Lartillot N, Philippe H (2008) Additional molecular support for the new chordate phylogeny. Genesis 46: 592-604.

21. Lartillot N, Philippe H (2008) Improvement of molecular phylogenetic inference and the phylogeny of Bilateria. Philos Trans R Soc Lond B Biol Sci 363: 1463–1472.

22. Zwickl DJ, Hillis DM (2002) Increased taxon sampling greatly reduces phylogenetic error. Syst Biol 51: 588-598.

23. Stefanovic S, Rice DW, Palmer JD (2004) Long branch attraction, taxon sampling, and the earliest angiosperms: Amborella or monocots? BMC Evol Biol 4: 35.

24. Lecointre G, Philippe H, Van Le HL, Le Guyader H (1993) Species sampling has a major impact on phylogenetic inference. Mol Phylogenet Evol 2: 205-224.

25. Philippe H, Laurent J (1998) How good are deep phylogenetic trees? Curr Opin Genet Dev 8: 616-623.

26. Lartillot N, Lepage T, Blanquart S (2009) PhyloBayes 3: a Bayesian software package for phylogenetic reconstruction and molecular dating. Bioinformatics 25: 2286-2288.
