# Supplementary material for: Resolving Difficult Phylogenetic Questions: Why More Sequences Are Not Enough
Source: PLoS Biol. 2011 Mar 15;9(3):e1000602. doi: 10.1371/journal.pbio.1000602 (PMC3057953; doi:10.1371/journal.pbio.1000602)
Supplement: Figure S1 — Phylogeny of the AT6 gene. (0.06 MB PDF) [file pbio.1000602.s003.pdf]

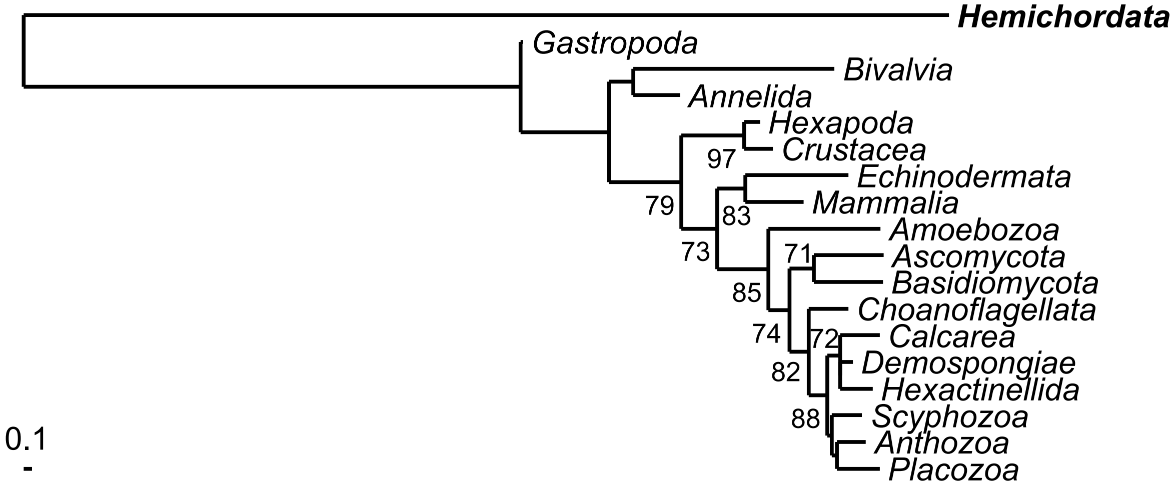

Figure S1: Phylogeny of the AT6 gene.

The alignment of Schierwater et al. was used to infer a single gene phylogeny with RAxML (LG+F+Γ model, 100 fast bootstrap replicates). The highly divergent sequence of Hemichordata (in bold) is non homologous.
